# How do high phosphate concentrations affect soil microbial communities after a century of ecosystem self‐reclamation?

**DOI:** 10.1111/1758-2229.70003

**Published:** 2024-10-23

**Authors:** Amandine Ducousso‐Détrez, Simon Morvan, Joël Fontaine, Mohamed Hijri, Anissa Lounès‐Hadj Sahraoui

**Affiliations:** ^1^ Université du Littoral Côte d'Opale, UR 4492 Unité de Chimie Environnementale et Interactions sur le Vivant (UCEIV) Calais Cedex France; ^2^ Institut de Recherche en Biologie Végétale (IRBV), Département de Sciences Biologiques Université de Montréal Montréal Quebec Canada; ^3^ African Genome Center Mohammed VI Polytechnic University (UM6P) Ben Guerir Morocco

## Abstract

The use of rock phosphate (RP) instead of soluble phosphate fertilizers is preferred for the development of more sustainable agriculture. However, the impact of high concentrations in RP on bacterial and fungal communities remains poorly documented. Thus, next‐generation sequencing was used to characterize bacterial and fungal communities in the soils and roots of four plant species growing naturally in a self‐restored ecosystem, on former open‐pit phosphate mines where past exploitation generated locally a substantial phosphate enrichment of the soil. Our results show that bacterial communities are dominated by Actinobacteria and Proteobacteria phyla, while the Ascomycota and Basidiomycota phyla predominate in the fungal community. The alpha and beta diversities of both bacterial and fungal communities differ significantly between the root and soil compartments but are not significantly affected by RP inputs. However, Amplicon Sequence Variants (ASVs) indicative of RP‐enriched soils have been identified; among them are bacteria representative of *Streptomyces*, *Bacillus*, *Mycobacterium* or *Agromyces*. Implications of these results open new ways of reflection to understand the microbial response following RP‐inputs and long‐term soil restoration, as well as to formulate microbial‐based bioinoculants for sustainable agriculture applications based on microorganisms better adapted to high concentrations of RP.

## Graphical abstract

We studied soil microbial communities in former phosphate mining areas, where substantial differences in soil phosphorus concentrations persist due to past mining activities. The results show striking similarities in microbiome patterns regardless of phosphorus concentrations. However, we identified indicator taxa for phosphorus‐enriched sites. We discussed the extent to which historical contingencies and microbial life strategies might explain these findings and proposed relevant avenues for formulating bio‐inoculants for agriculture or land reclamation.

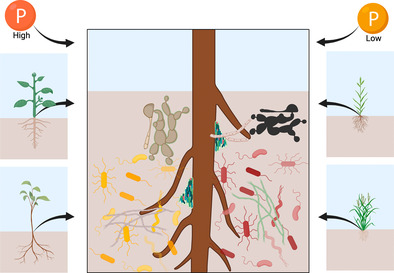

## INTRODUCTION

Phosphate fertilization as soluble phosphorus (P) has been used in agricultural systems to contribute significantly to current global food production and security. Indeed, even when the soil has high levels of total P, only a small fraction of it is actually available for uptake by cultivated plants. Some authors estimate it may be <1% (Bünemann, 2015; Rai et al., 2013). This low P availability is a major productivity constraint in many natural or managed ecosystems. In order to overcome this constraint, the soils are enriched with soluble orthophosphate ions through soil amendments for decades. P‐fertilization is then mainly applied using chemical inputs derived from phosphate‐rich rock deposits, then industrially processed into marketable products. However, currently global reserves of high‐quality rock‐phosphate (RP) are reaching alarmingly low levels (Cordell et al., 2009; Cordell & White, 2011, 2015; George et al., 2016; Reitzel et al., 2019). In addition, anthropogenic activities have altered the global P biogeochemical cycle and cause global environmental, social, economic and geopolitical challenges. In particular, environmental damages have emerged, such as water eutrophication or high accumulation of legacy P fixed to soil solid phase (Buczko & Kuchenbuch, 2007; Zhu et al., 2018). Consequently, to avoid a potential global P crisis, a variety of strategies are invested. Among the potential strategic options, the valorization of soil resources and processes involved in soil P dynamics appears promising. Indeed, in soils, total P‐pool includes inorganic and organic P compounds. The chemical speciation and their contribution to the soil supply in soluble available P‐forms are driven by a set of physical, chemical and biological soil factors (Chen et al., 2021; Ducousso‐Détrez, Fontaine, et al., 2022a; Hu et al., 2022). In particular, numerous microorganisms are able to convert insoluble P compounds into bioavailable P‐forms, thus facilitating P‐uptake by plant roots. These microorganisms are referred to as phosphate‐solubilizing microorganisms. This functional group is extensively and diversely studied (Alori et al., 2017; Kafle et al., 2019; Kalayu, 2019; Kishore et al., 2015; Kour et al., 2021; Raymond et al., 2021; Rodríguez & Fraga, 1999; Sharma et al., 2013; Tian et al., 2021). It includes a wide range of organisms, among them being soil phosphate‐solubilizing bacteria (Batool & Iqbal, 2019; Elhaissoufi et al., 2020; Gómez‐Muñoz et al., 2018; Liu et al., 2020; Mpanga et al., 2018) and phosphate‐solubilizing fungi (Efthymiou et al., 2018; Elias et al., 2016; Leggett et al., 2015; Raymond et al., 2018).

Concomitantly, the agronomic relevance of phosphate‐solubilizing microorganisms for enhancing plant performance and agricultural yield has been proved. Soil inoculation with these microorganisms can reduce P fertilizer application rate by 50% without significant reduction of the crop yield (Jilani et al., 2007; Rafi et al., 2019). Thus, different types of microbial inoculum have been developed as P bio‐fertilizer (Bashan et al., 2014; Hijri, 2016; Lawson et al., 2019). However, some uncertainties in the efficiency of inoculants in field applications are often underlined (Bargaz et al., 2018). The concept of phosphate‐solubilizing bacteria and the production of inoculants based on such microorganisms for increasing crop P nutrition are also being questioned. For instance, Raymond et al. (2021) have extensively discussed whether microbial P solubilization ability is a trait linked to specific groups of soil taxa or a general property of the overall soil microbial community. It is essential to decipher microbial patterns in P‐rich soils in order to understand the microbial response to P‐inputs and fertilization, adapt cultural management practices and formulate microbial inoculants adapted to P‐rich soils, which are relevant for improving plant growth and crop culture.

Thanks to the progress of the next generation sequencing technologies, several studies provide evidence that contrasting P fertilization regimes (i.e., organic vs. inorganic P sources, P‐concentration) shape the microbial communities in interaction with plants (Fabiańska et al., 2019; Gomes et al., 2018; Robbins et al., 2018; Silva et al., 2017; Yu et al., 2018). Previous studies on the effects of P supplementation on soil microbial communities have been conducted using a variety of experimental setups. Thus, researchers have investigated microbial communities in various environments, including fields (Gomes et al., 2018; Silva et al., 2017), forests (Liu et al., 2012), tree plantations (Huang et al., 2016), pastoral systems (Wakelin et al., 2012), and under controlled environmental conditions (Bodenhausen et al., 2019; Fabiańska et al., 2019; Robbins et al., 2018). They have also analysed different plant species and genotypes, as well as different soil compartments, such as bulk soil and root‐associated compartments like the rhizosphere, rhizoplane and root endosphere (Fabiańska et al., 2019; Gomes et al., 2018; Robbins et al., 2018; Silva et al., 2017; Yu et al., 2018). Some studies have focused on the impacts of exogenous P amendments on microbial communities following relatively short‐term P fertilization regimes (Bodenhausen et al., 2019; Fabiańska et al., 2019; Gomes et al., 2018; Gumiere et al., 2019; Ikoyi et al., 2018; Trabelsi et al., 2017). Others have examined soil microbiome responses after repetitive and consecutive pulses of P availability over decades of fertilization processes (Francioli et al., 2016; Huang et al., 2016; Leff et al., 2015; Liu et al., 2012; Robbins et al., 2018; Silva et al., 2017; Tang et al., 2016; Wakelin et al., 2012; Wang et al., 2016; Yu et al., 2018). In most studies, P is generally supplied at P levels close to or slightly above plant requirements, and the microbial response observed is a snapshot of the effect of a recent P resource amendment, even after a long‐term fertilization process. Researchers have also investigated the role of different P sources in shaping microbial communities, varying P levels (Bodenhausen et al., 2019; Gomes et al., 2018), and various chemical forms of P, including industrial processed fertilizers, RP, nutrient solutions, and manure.

The impact of RP has been notably analysed (Gumiere et al., 2019; Pattanayak et al., 2007; Silva et al., 2017; Trabelsi et al., 2017). Indeed, the use of raw RP has been proposed in different agricultural systems as an alternative to reduce the use of industrial fertilizers (Cordell et al., 2009; Richardson & Simpson, 2011; Van Kauwenbergh, 2010; Zapata & Roy, 2004). In addition, although RP has a lower reactivity than commercial fertilizers when applied directly in the field, P availability from these rocks can be increased through the action of the soil microbiota. Consequently, RP has been targeted as an effective agronomic product when applied directly to soils, in combination with microbial phosphate solubilizing inoculum, to promote crop production by improving plant P acquisition (Kaur & Sudhakara Reddy, 2017; Manzoor et al., 2017; Soltangheisi et al., 2018).

Thus, the present study aims at investigating the patterns of native bacterial and fungal communities across natural sites inside a mining area, expected to be enriched in RP deposits at various grades, due to localized excavation of crude ores by past mining activities. Using sequencing amplicons of the 16S rRNA gene and the ITS region, we characterized the indigenous bacterial and fungal communities in the roots and rhizospheric soil of native plant species (*Ranunculus bulbosus* L., *Bromus sterilis* L., *Taraxacum officinale* F.H. Wigg. and *Dactylis glomerata* L.), across soils “with” or “without” excavated RP ore inputs. Microbial diversity was evaluated in the different sampled soils using alpha and beta diversity indices. We then explored microbial community composition at different taxonomic levels. Indicator species analysis was also performed among communities of the different soils. The ecological significance of the microbial profiles observed is discussed in link to microbial response to RP‐rich soils and ecosystem restoration after mining perturbations. The implications of the results for formulating microbial‐based bioinoculants for sustainable agriculture applications based on more adapted microorganisms to high concentrations of RP are also discussed.

## EXPERIMENTAL PROCEDURES

### Study area

The study was conducted in natural sites, located in the geographical area of the “Phosphatières du Quercy” (44°22′22″N, 1°41′16″E) near Bach, southwestern France. In this area, fillings of phosphorites, a phosphatic ore (containing up to 38% P_2_O_5_; Bornuat, 2009) naturally stored in the clay of limestone plateaus of the Quercy palaeokarst, were exploited from 1870 to 1907 to produce P‐fertilizers. Locally, due to mining practices, one‐time ore inputs of excavated crude RP have been abandoned on the topsoil. Consequently, strips of strong ecologically disturbed soils (P‐soils), enriched one century ago by excavated rocks including sedimentary phosphorite (e.g., deep soil layer brought in the surface as topsoil), closely co‐occurred side by side with native, non‐disturbed soils (nP‐soils). After this single pulse of P nutrients from RP inputs, these mine lands were abandoned, re‐colonized by spontaneous vegetation, and naturally revegetated over the course of a century, with no significant human disturbance until today.

**FIGURE 1:**
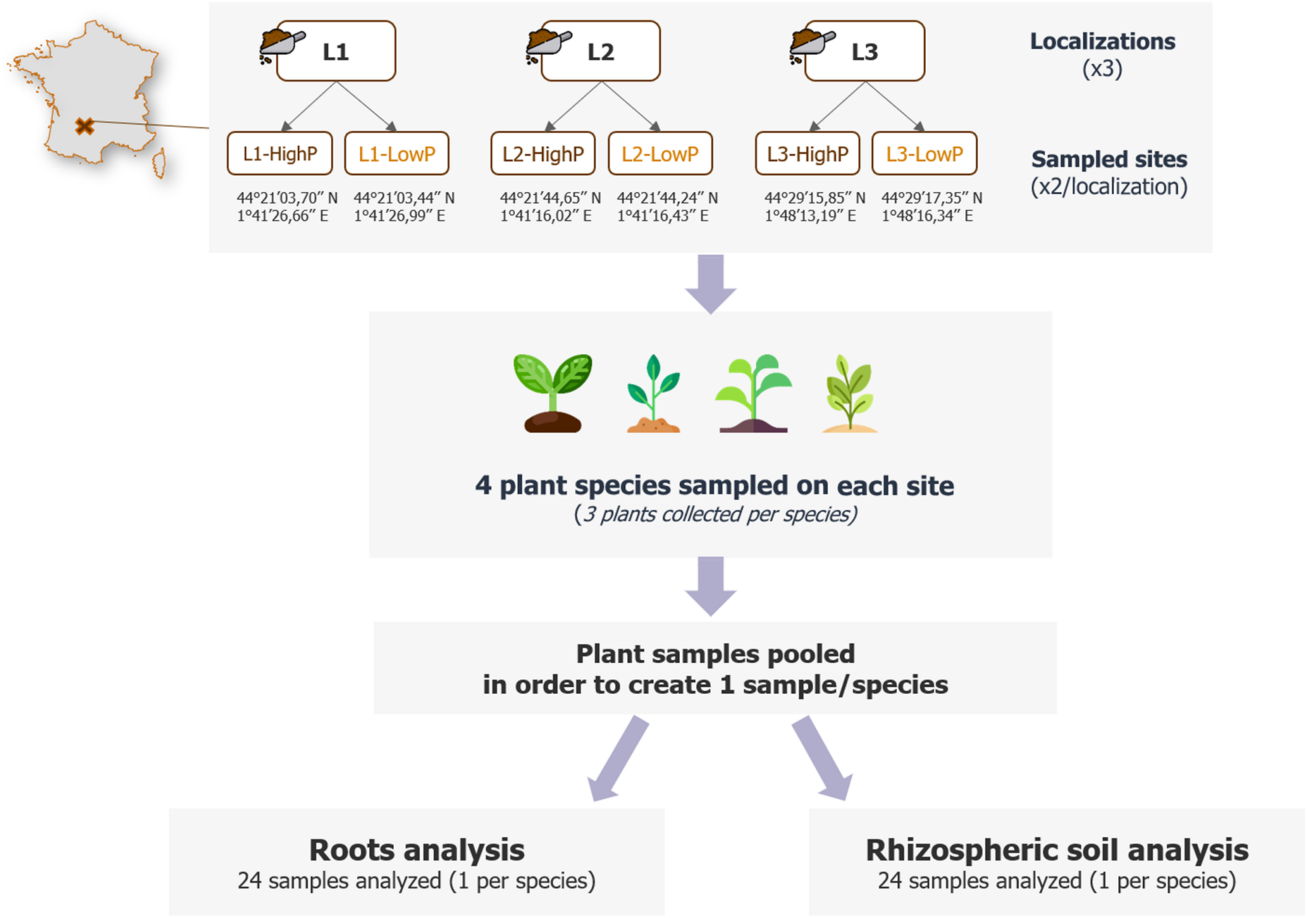
Study area and experimental setup.

We selected three old mines in the Phosphatières area: Cloup d'Aural (L1), Valbro (L2) and Mémerlin (L3). In each localization, two site profiles have been investigated: an undisturbed native site “without” ancient mining inputs (hereafter referred as nP‐soil), and a well‐established adjacent disturbed site “with” mining inputs of excavated phosphorite rocks (hereafter referred as P‐soil). Thus, six sites (2 paired sites per location) were identified and geo‐referenced (Figure 1).

### Plant sampling

In the six referenced sites, a set of plant species was sampled, namely *Ranunculus bulbosus* L., *Bromus sterilis* L., *Taraxacum officinale* F.H. Wigg. and *Dactylis glomerata* L. These species were chosen as they correspond to the native herbaceous plants that are sufficiently abundant in the floristic processions of the six sites. Furthermore, they are referenced in the scientific literature as mycorrhizal species (Wang & Qiu, 2006). Additionally, two of the species are monocotyledonous (*Bromus sterilis* L. and *Dactylis glomerata* L.) and two are dicotyledonous (*Ranunculus bulbosus* L. and *Taraxacum officinale* F.H. Wigg.). Finally, with the exception of *Bromus sterilis* L., which is an annual species, all of the other species sampled are perennials.

For each species, three individual plants were collected from each of the six studied sites on 29 January 2019. All plants were sampled at a vegetative stage and 5–20 cm of soil was collected with each plant and quickly transported to the laboratory in coolers. A total of 72 plants were harvested, and the mycorrhizal colonization rates were determined. Percentages ranging from 13 to 63 were obtained, as previously reported (Ducousso‐Détrez, Raveau, et al., 2022b), depending on the sampling site and the plant species.

### Separation of root and rhizospheric soil samples

Two distinct fractions across the soil–plant continuum were harvested: (i) root samples and (ii) rhizospheric soils closely associated with plant roots, referred as “soil” samples.

Each root sample was a composite of root fragments, collected from three plants per species and then pooled. The soil attached to the roots was removed by gentle agitation, and then the roots were washed in distilled water. Thus, four root samples per site were prepared, resulting in a total of 24 root samples for all six sites. For each root sample, one fraction was frozen at −20°C before subsequent molecular analysis.

In the same manner, each soil sample was a composite of soils collected from the three plants of the same plant species and subsequently pooled. The root fragments were removed from these samples. These 24 rhizospheric soil samples were then immediately stored at −20°C until further DNA extraction.

### Soil physico‐chemical properties characterization

For each site, several soil fractions that were not adhering to plant roots were collected and then pooled to obtain a composite bulk soil sample. These samples were then characterized by analysing the total phosphorus and available Olsen phosphorus concentrations. To complete the study, granulometry (clays, silts and sands), pH, and total chemical element content (performed primarily by inductively coupled plasma atomic emission spectroscopy/ICP‐AES) were determined by the CIRAD‐US Analyse laboratory (Montpellier, France). Additionally, an X‐ray fluorescence analysis (XRF Titan 800 S X‐Ray Fluorescence) was performed on soil capsules pressed at 20 T for 2 min, to evaluate the concentrations of elements ranging from Mg to U.

The full analysis data have been previously provided in Ducousso‐Détrez, Raveau, et al. (2022b). Briefly, pH values ranging from 6.9 to 7.3 were obtained, and no significant differences between sites were found. Comparing the paired sites (i.e., P vs. nP site) from the same mining area, significant differences were observed for P and Ca contents between the P and nP soils. P‐soils had total P contents ranging from 2860 to 13,928 mg kg^−1^ while nP‐soils ranged from 1067 to 1496 mg kg^−1^ (Table 1). Also, clear differences in terms of available Olsen P levels were identified (Table 1), with higher concentrations in P‐soils (ranging from 46.1 to 339.5 mg kg^−1^) compared to nP‐soils (ranging from 5 to 12.8 mg kg^−1^). Thus, the available‐P fractions ranged from 1.61 to 2.43% of the total P content in P‐soils; they did not exceed 1% of total P in nP‐soils (0.36 to 0.85%), in accordance with the literature (Barber, 1995; Bieleski, 1973; Rodríguez & Fraga, 1999).

**TABLE 1:** Total and available P concentrations (mg kg^−1^) in the six sampled sites (from Ducousso‐Détrez, Raveau, et al., 2022b).

Localizations	L1		L2		L3	
Sites	nP	P	nP	P	nP	P
Total P	1380	2860	1067	13,928	1496	10,739
Available P (Olsen P)	5	46.1	8.1	339.5	12.8	192.8
Ratio: available P/total P (in %)	0.36	1.61	0.76	2.43	0.85	1.79

The XRF analysis highlighted the presence of trace elements such as chromium (176–227 ppm), nickel (38–52 ppm), arsenic (23–42 ppm), and lead (35 to 50 ppm), which are typically found in RP as minor constituents. Metal trace element contaminants such as cadmium, mercury, and vanadium were below the limit of detection of our equipment. However, no significant differences between P‐soils and nP‐soils were found for these trace elements.

### DNA extraction from soils and roots

From the soil samples, total genomic DNA was extracted in triplicates (24 soil samples, 250 mg of soil per sample) using Nucleospin Soil R kit (Macherey‐Nagel, Düren, Germany), according to the manufacturer's instructions.

Genomic DNA from roots was extracted in triplicate using a method adapted from Abu‐Romman (2011) and Aleksic et al. (Aleksić et al., 2012). Briefly, roots were frozen in liquid nitrogen (−196°C) in a sterilized mortar and ground into fine powder. The ground roots (300 mg) were then subject to Cetyl trimethyl ammonium bromide (CTAB—1.4 M NaCl, 100 mM Tris–HCl pH 8.0, 20 mM EDTA pH 8.0, 2% CTAB), Polyvinylpyrrolidone (PVP 1% w/v), ß‐Mercaptoethanol (5% v/v) and activated charcoal (0.5% w/v) extraction (30 min.; 55°C). After this incubation period, a centrifugation step was carried out (10 min.; 16,000 g) and the lysate extraction was then performed using two successive steps with chloroform: isoamylalcohol (24:1). DNA precipitation then occurred in the presence of isopropanol (1 h incubation; 25°C), followed by another centrifugation (10 min.; 700 g). The DNA pellet was then washed three times in a row by addition of ice‐cold ethanol (70%) and centrifuged (10 min.; 900 g), before air drying at room temperature (~90 min.; 20°C). Finally, the DNA pellet was dissolved in 50 μl of TE buffer (10 mM Tris–HCl, pH 8.0; 1.0 mM EDTA, pH 8.0; Ducousso‐Détrez, Raveau, et al., 2022b).

For both types of samples (root or soil), the quality of the extracted DNA was performed using 1% (w/v) agarose gels. Quantification of extracted DNA was carried out on a Spectra Max R iD3 spectrophotometer (Molecular Devices LLC, Sunnyvale, CA, United States). The concentration of all samples was determined, and DNA extracts were diluted to 25 ng.ml^−1^ for further analyses. The extracted DNA was stored at −20°C until use.

### PCR reactions, amplicon library generation and sequencing

In order to profile the bacterial communities, we used the primer pair 341(F): 5′‐CCTACGGGNGGCWGCAG‐3′, and 805(R): 5′‐GACTACHVGGGTATCTAATCC‐3′ targeting the V3–V4 hypervariable region of the bacterial 16S ribosomal RNA (rRNA) gene, with an expected size of the amplicon of around 400 base pairs (bp), in length (Herlemann et al., 2011; Mizrahi‐Man et al., 2013; Morvan et al., 2020). The PCR amplification were performed with reaction mixtures (25 μl) contained 5 μl of Q5 (5×) reaction buffer and 0.25 μl (2 U μl^−1^) of Q5 High‐Fidelity DNA Polymerase (New England Biolabs France, Évry, France), 0.8 μl of forward and reverse primers (0.4 μM), 1 μl of dNTPs (0.2 mM), 1 μl of DMSO, 1 μl of Bovine Serum Albumin (BSA; 100 μg ml^−1^) and 1 ng of environmental DNA template. The thermal profile used in order to obtain bacterial rDNA amplicons was as follows: preheating 95°C for 3 min. of initial denaturation, followed by 35 cycles at 95°C for 30 s, annealing at 56°C for 30 s, extension at 72°C for 50 s and final extension step at 72°C for 5 min. (Raveau et al., 2020).

Regarding the fungal communities, the PCR amplification targeted the internal transcribed spacer (ITS) segments located between the 5.8S and LSU genes of the ribosomal RNA, using the primers ITS3_KYO2 (F): 5′‐GATGAAGAACGYAGYRAA‐3′, and ITS4_KYO3 (R): 5′‐BTTVCCKCTTCACTCG‐3′ (Toju et al., 2012). Although this region can vary in length (Sietiö et al., 2018), the primer pairs ITS3_KYO2/ITS4_KYO3 generate amplicons of around 430 bp (Toju et al., 2012). The reaction mixture (25 μl) included 5 μl of Q5 (5X) reaction buffer and 0.25 μl of Q5 High‐Fidelity DNA Polymerase (New England Biolabs France, Évry, France), 0.8 μl of forward and reverse primers (0.4 μM), 1 μl of dNTPs (0.2 mM), 1 μl of DMSO, 1 μl of BSA (100 μg ml^−1^) and 1 ng of environmental DNA template. The thermal cycling conditions for preparation of fungal ITS rDNA amplicon libraries were performed with an initial denaturation for 5 min. at 95°C, followed by 35 cycles of 94°C for 20 s, 47°C for 30 s and 72°C for 20 s, and with a final extension step at 72°C for 7 min. (Raveau et al., 2020).

All primers used for PCR amplification were coupled with CS1 (5′‐ACACTGACGACATGGTTCTACA‐3′) and CS2 (5′‐TACGGTAGCAGAGACTTGGTCT‐3′) adapters at the 5′ end. All the amplification reactions were conducted with PCR thermocycler (Agilent Surecycler 8800) and quality control for band size of the amplicons was operated on 1.5% agarose gel electrophoresis before sequencing of libraries.

The triplicate of each amplified PCR samples with adapters were pooled together and sent to the Genome Quebec Innovation Centre (Montreal, QC, Canada) for barcoding and sequencing using an Illumina MiSeq sequencer producing paired‐end reads of 2 × 300 bp in length.

### Bioinformatic processing for sequencing data analysis

Bioinformatic analyses were performed using the R 4.0.2 software (R Core Team, 2019). For metabarcoding analysis of bacterial and fungal communities, sequences were clustered using the Amplicon Sequence Variant (ASV) approach based on cluster generation using a 100% similarity threshold, while operational taxonomic units (OTUs) are based on 97% similarity threshold for sequence identity and key taxonomic groups sequences. Minimizing the risk of diversity loss in ASV clustering aligns with our goal of achieving a finer level of taxonomic resolution. By reducing bias, we can more accurately capture species identity and detect subtle differences between microbial communities, especially when using indicator species analysis (Rolling et al., 2022). The DADA2 pipeline (v. 1.16) was used to process the different sets of paired‐end sequences of each gene data set (Callahan et al., 2016). Following the visualization of quality profiles, a filtration step of reads was performed to eliminate poor quality sequences, non‐merging sequences as well as chimeric sequences. Subsequently, the ASVs obtained from the DADA2 pipelines were filtered for very low‐abundance ASVs before any further analysis: sequences present only once (singletons) or twice (doubletons) in the whole data set were eliminated across the microbial profiles (Wen et al., 2017). Rarefaction curves, which show the observed ASVs numbers as a function of sequencing effort, were computed with the function ‘rarecurve’ from the Vegan R package to estimate if sequencing depth was sufficient to capture the whole diversity present. ASV count tables were generated using usearch_global from USEARCH package and the taxonomic assignment was carried out using the assignTaxonomy() function which implements the RDP naive Bayesian classifier method described in Wang et al. (2003). The Silva v132 database formatted for the DADA2 (Callahan et al., 2019) was used to assign bacterial 16S rRNA gene sequences from kingdom to genus (minimum bootstrap 80). The UNITE web‐based database was used to assign fungal taxa (Kõljalg et al., 2013).

The gene sequences of the whole dataset have been deposited in NCBI Sequence Read Archive (SRA) database and can be found under the project accession number PRJNA784523.

### Microbial community analysis

Statistical analyses (R 4.0.2 software, R Core Team, 2019) were run independently for bacterial and fungal communities. The impacts of compartmentalization (root vs. soil) and P status (P‐soil vs. nP‐soil) were tested; comparisons were performed either between samples of the same localization or by pooling localizations together and comparing soil P contents.

#### Diversity measures: Alpha and beta diversity statistics

The local alpha diversity (Whittaker, 1972) was estimated in each sample by computing the Chao1, Shannon and Simpson statistics (Kim et al., 2017; Shannon, 1948; Simpson, 1949) from the plot_richness() function from the phyloseq package (McMurdie & Holmes, 2013). No read depth normalizing or supplementary rarefaction were performed to capture more carefully the diversity and keep a maximum of ASVs. The sample data were graphed using ggplot2 to obtain boxplot (Wickham & Wickham, 2016). Ranks of the alpha diversity indices were subjected to ANOVA test with the significance determined by a permutation test using the aov() function of the Vegan package (Oksanen et al., 2018), followed, if required, by a post hoc Tukey's Honest Significant Difference (HSD) test (*p* <0.05) allowing pairwise comparisons to assess the effect of plant compartment and soil RP contents.

Beta diversity, which is the variations in microbial community structure among samples, was measured and visualized by principal coordinate analysis (PCoA) based on the Hellinger distances as dissimilarity measure. Permutational multivariate analysis of variance (PERMANOVA) was performed to compare statistically the clusters of samples and to test for significant differences among centroids, using adonis2 function in the Vegan package (significance tested by 9999 permutations). In addition, Permutest.betadisper function was used to consider the multivariate homogeneity of group dispersions (i.e., distance of group members to the group centroid) when interpreting the results of PERMANOVA. The statistical significance for all tests was set at *p* values <0.05.

#### Variations in microbial taxonomic composition

The R package *metabarcoder* was used to parse, manipulate and visualize in a tree format using taxonomic classification, how samples vary in their taxonomic composition. Thus, to display differences in abundance for each taxon, at every taxonomic rank, between two sample groups, the compare groups() function in metacoder package was used after calculating the per‐taxon abundance from the ASV read counts with the calc_taxon_abund() function. The Wilcox rank‐sum test was computed to test for significance of difference between the median abundances of samples in each treatment followed by a Benjamini‐Hochberg (FDR) correction for multiple comparisons. For visualization, the standard heat_tree() function was used to compute differential heat trees to depict statistics quantitatively associated with taxa and display, across pairwise comparisons, in which sample each taxon is more abundant in, using the colour and the size of nodes and edges in a taxonomic tree coloured with a diverging colour scale (Foster et al., 2017; Iburg et al., 2021). The statistic used for plotting was the log2 of the ratio of median proportion of reads in the two samples groups compared.

#### Indicator species analysis

The indicator taxa are defined based on a combination of specificity (occurring in that environment more frequently than other environments) and fidelity (the majority of taxon members are found in that environment) as described by Legendre and Legendre (2012). The multipatt() function from indicspecies package was used on the ASV abundance data table (de Cáceres & Legendre, 2009). An indicative species value was assigned to prospective indicative species based on their importance in the group they are found to be indicative of. The IndVal's p_values with the p.adjust() function was computed from 9999 permutations (significant at the *α* = 0.05 level).

## RESULTS

### Descriptive results of bioinformatic data

To characterize the fungal and bacterial communities in the rhizospheric soil and root samples across the six sites, the metabarcoding approach via Illumina MiSeq sequencing resulted in a total of 4,890,709 reads for the bacterial 16rRNA gene and 3,642,907 reads for the fungal ITS region over the sample set (i.e., 24 soil samples and 24 root samples; Table S1). Following the visualization and correction of the quality profile plots of reads with DADA2's filtering function, dereplication then merging of sequences, a total of 6720 ASVs were inferred from the 16S rRNA gene data. The ITS data were clustered into 6664 ASVs (Table S1). Information about sequencing depth for both 16S rRNA and ITS gene data are available from rarefaction curves for each sample (Figures S1 and S2).

### Alpha diversity in P‐soils and nP‐soils and in rhizospheric versus root compartment

**FIGURE 2:**
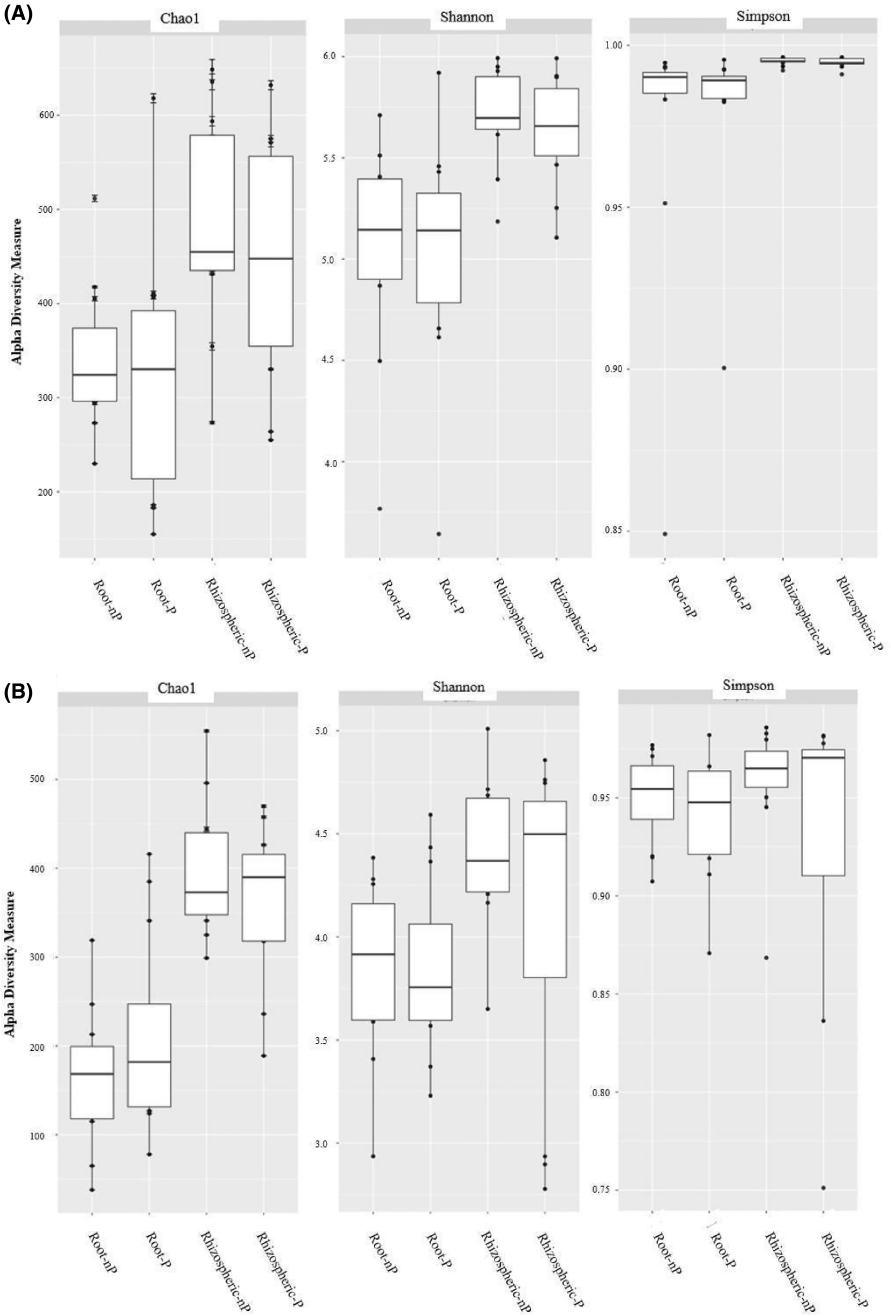
Alpha diversity of the over‐all 16S rRNA bacterial gene communities (A) and the over‐all fungal ITS communities (B) communities, as measures by richness (number of ASVs, Chao1, richness Shannon (bacteria *p* value = 7.126e^−05^; fungi *p* value = 0.0899 and Simpson indices (bacteria *p* value = 0.117; fungi *p* value = 0.472), within rhizospheric soil and root compartment sampled in P and nP sites. P: With mining RP ore deposit; nP: Without mining RP ore deposit.

Computing the Chao1, Shannon and Simpson alpha‐diversity metrics, we compared microbial diversity according to the plant‐associated compartments (root vs. rhizospheric soil) and according to the RP status (nP‐soils vs. P‐soils) across the six sampled sites (Table S2). Overall, the results showed that fungal alpha diversity values were generally lower than the bacterial ones within each compartment, no matter the metrics (Table S2). Besides, the Shannon and Simpson indices of bacterial communities were not significantly impacted by RP status (Figure 2A). Similarly, fungal diversity indices did not differ significantly comparing P‐soils and nP‐soils (Figure 2B).

In contrast, significant variations in alpha diversity indexes were observed across the rhizospheric soil relative to the root‐compartment. For the bacterial communities, higher Shannon and Simpson values were significantly noticed in rhizospheric soil, performing global analysis. However, Simpson metrics which are known to give less weight to rare ASVs, were not significantly affected by plant compartment effect. Moreover, analysis of the fungal dataset at local level showed that the Shannon indices were significantly influenced by the nature of the compartment in L1 localization (data not shown).

### Beta diversity in P‐soils and nP‐soils and in rhizospheric versus root compartment

**FIGURE 3:**
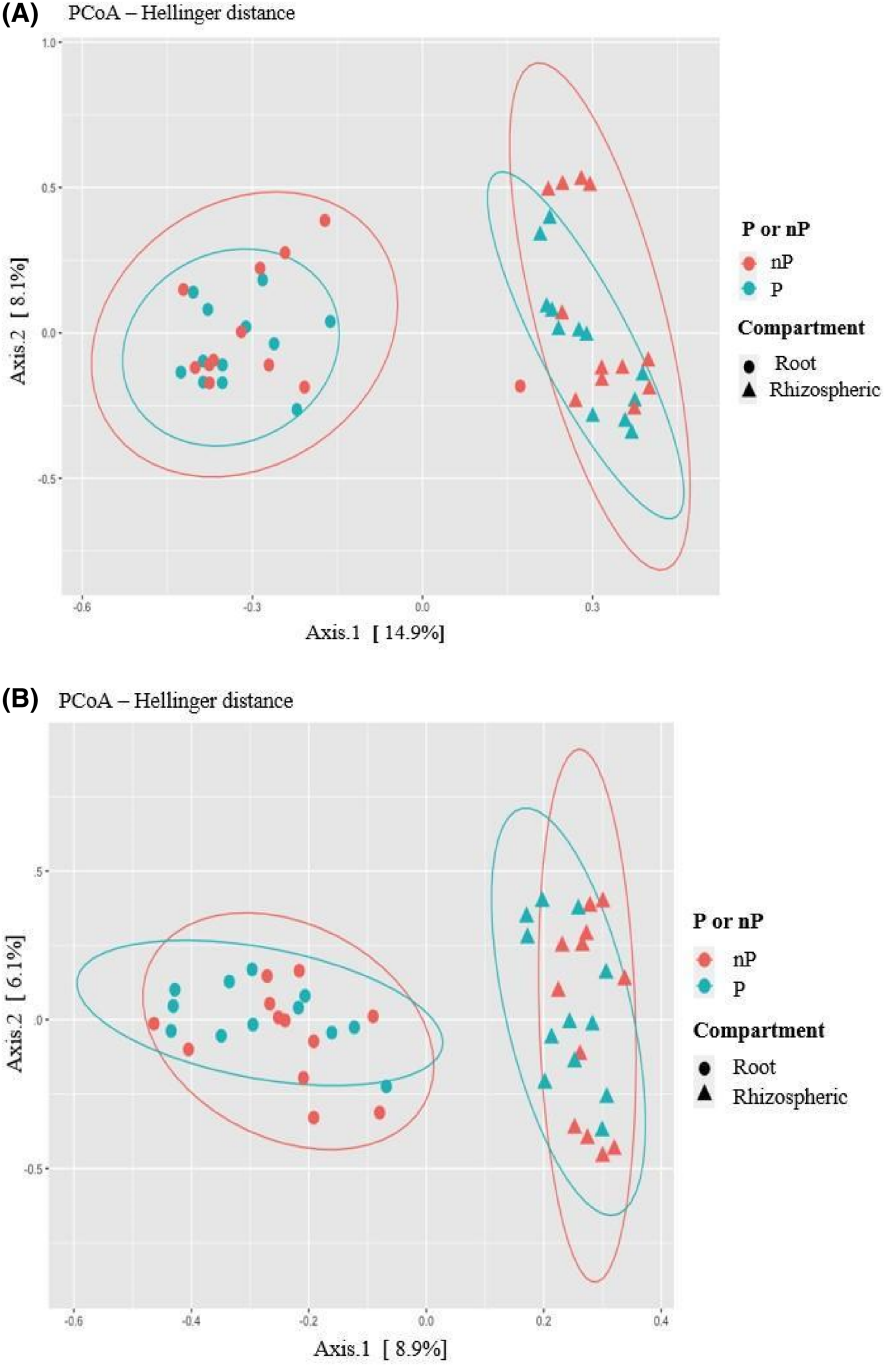
Beta diversity analysis among the over‐all data set: PCoA ordination, based on Hellinger distances, of bacterial 16S rRNA gene sequence data (A) and fungal ITS sequence data (B). Analysis across the over‐all data set, that is, L1‐P/L2‐P/L3‐P samples pooled together versus L1‐nP/L2‐nP/L3‐nP (48 samples for each microbial community; each coloured symbol represents a sample).

The PCoA ordination plot representing bacterial beta diversity clearly shows a distinction between two clusters along the first axis, corresponding to either soil or root samples (Figure 3A). Additionally, the PERMANOVA test indicated a significant effect of the compartment on the bacterial beta diversity.

A similar segregation pattern was observed for fungal beta diversity but the variation fraction attributable to compartment did not exceed 8.9% (Figure 3B).

**FIGURE 4:**
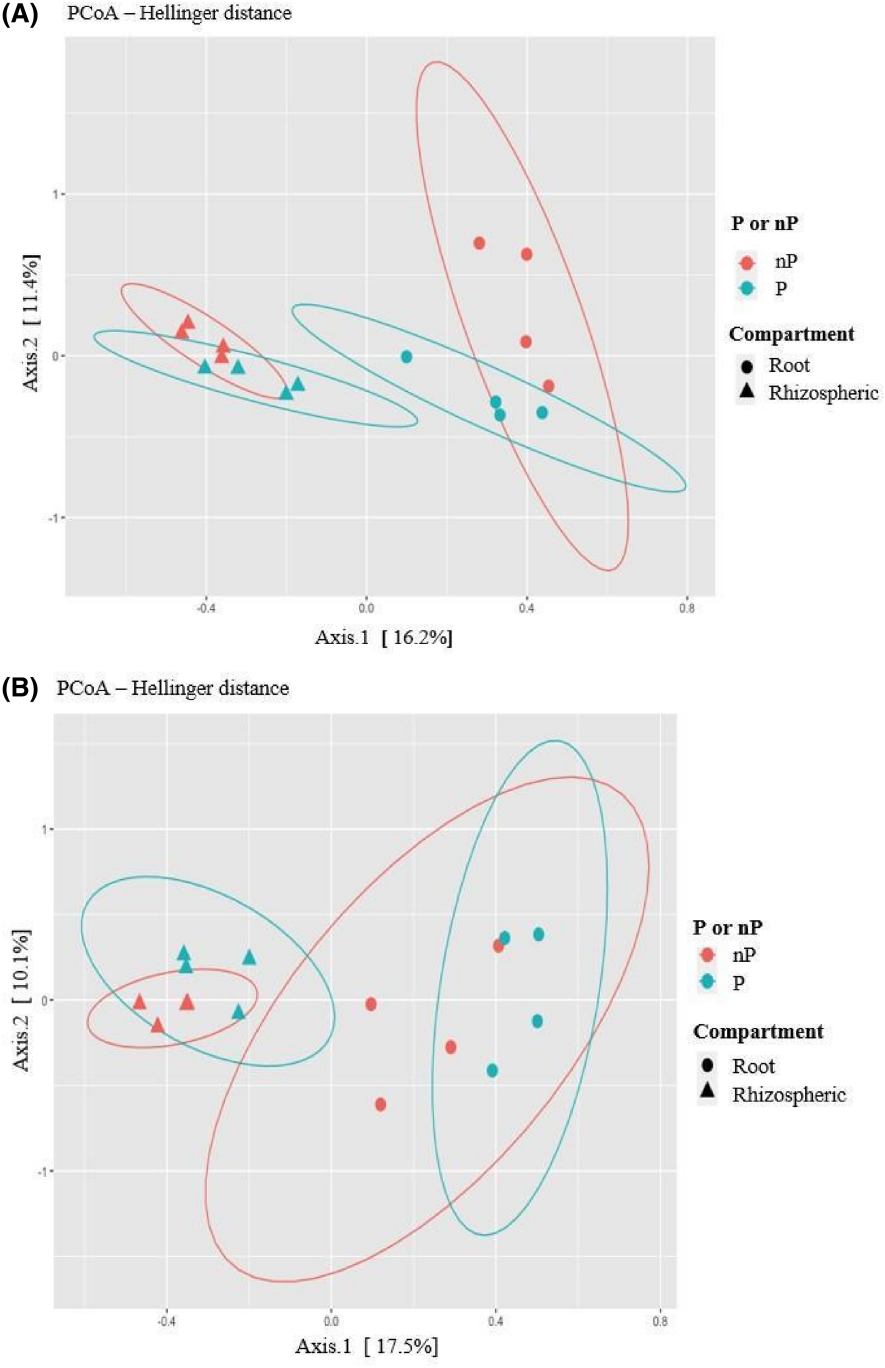
Beta diversity analysis in pairwise soil comparisons: PCoA ordination, based on Hellinger distances, of fungal ITS sequence data when comparing (A) L1‐P versus L1‐nP soils within L1 localization (8 samples), (B) comparison L2‐P versus L2‐nP soils within L2 localization (8 samples). P: With mining RP ore deposit; nP: Without mining RP ore deposit.

Next, we investigated if a fraction of beta diversity was attributable to soil P‐status. Indeed, a significant effect of P‐status on bacterial and fungal communities clustering was observed when assessing its effect from the over‐all data set (respectively, *p* value = 0.024 and *p* value = 0.0168). In contrast, when comparing matched soils from the same location, the assessment of its impact on fungal community diversity was significant in L1 and L2 (Figure 4A,B), but not in L3. Furthermore, P‐status did not have a significant effect on the bacterial community's ordination, when comparing matched soils, regardless of location (data not shown).

### Global taxonomic composition of all the bacterial and fungal communities

**FIGURE 5:**
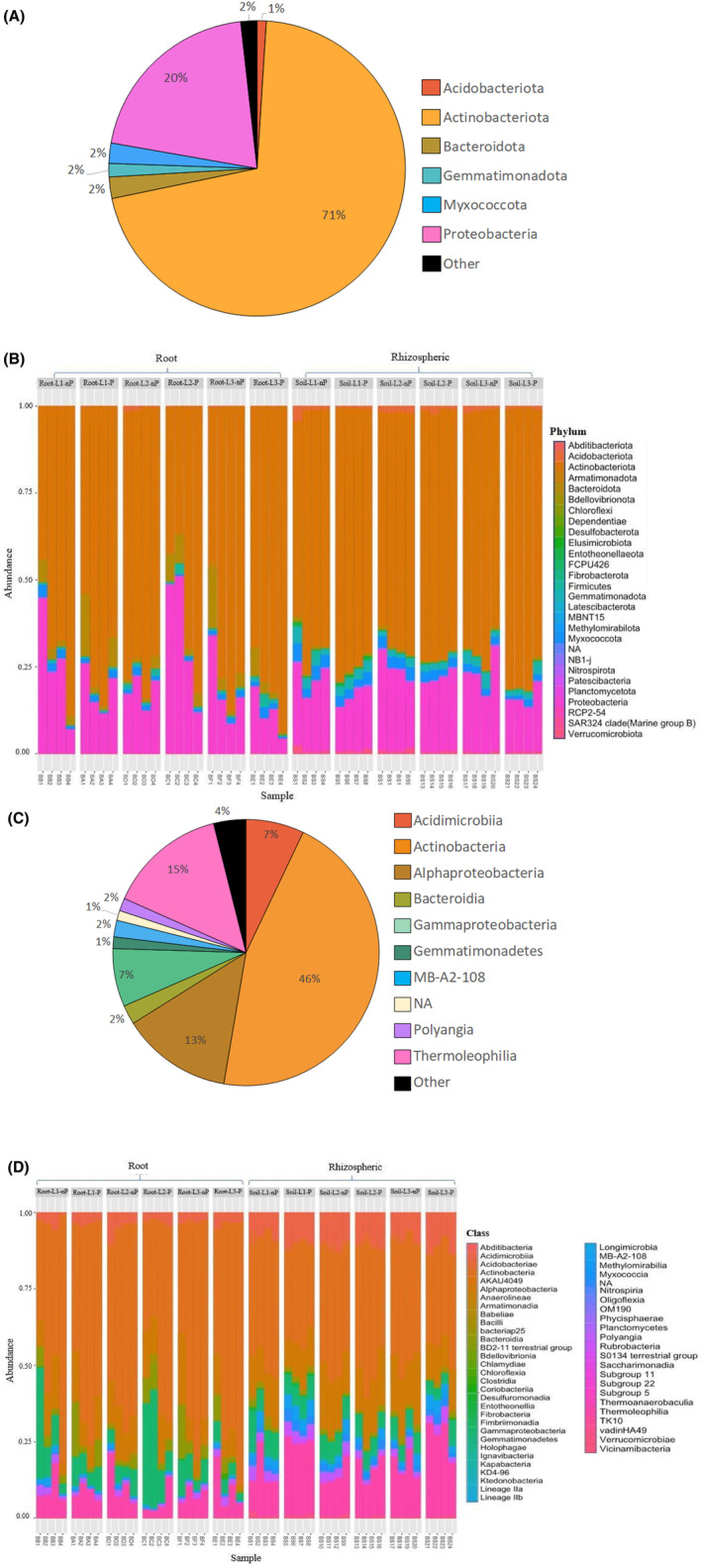
Taxonomic distribution patterns of the bacterial population at phylum (A, B) and class levels (C, D). A and C: Distribution of the global bacterial community (overall populations from the 48 samples pooled) at phylum level (A) and across the major classes (i.e., more than 1% of total reads) (C). B and D: Relative abundance profile (percentage of total 16S rRNA gene sequences) at the phylum level (B) and across the major classes (D) of bacterial 16S rRNA gene amplicon data, for each soil and root samples at each sampled site. Relative abundance was computed across the overall bacterial data set. “NA” category: ASVs that did not obtain taxonomic assignment at phylum or class level; “Other” category: Mix of taxa with low abundance (<1%).

From all the bacterial ASVs obtained across the overall samples (48), 28 bacterial phyla were identified; 15% of the ASVs were non‐assigned (NA) to a phylum. Based on the relative read counts (Figure 5A,B), the prevalent phyla were Actinobacteriota (71%) and Proteobacteria (20%). Bacteroidota, Myxococota and Gemmatimonadota phyla were also identified, but are less abundant (<2.5%), as well as Acidobacteriota, Firmicutes, Verrucomicrobiota and Entotheonellaeota (<1% each). A total of 54 classes were recovered and among them, six are dominant: Actinobacteria is the major class (46%) while Thermoleophilia and Alphaproteobacteria reached around 15% and 13%, respectively; Acidimicrobiia and Gammaproteobacteria accounted for 7% each (Figure 5C,D). The other classes reached <3% and the reads NA to a class represented about 1%. Among the 126 orders recognized, the most abundant are Micromonosporales (15.9%), Rhizobiales (8.5%), Solirubrobacteriales (8.3%), Propionibacteriales (7.9%), Gaiellales (5.7%) and Streptomycetales (5.2%). Non‐assigned ASVs at the order level accounted for 4.75% of the reads.

**FIGURE 6:**
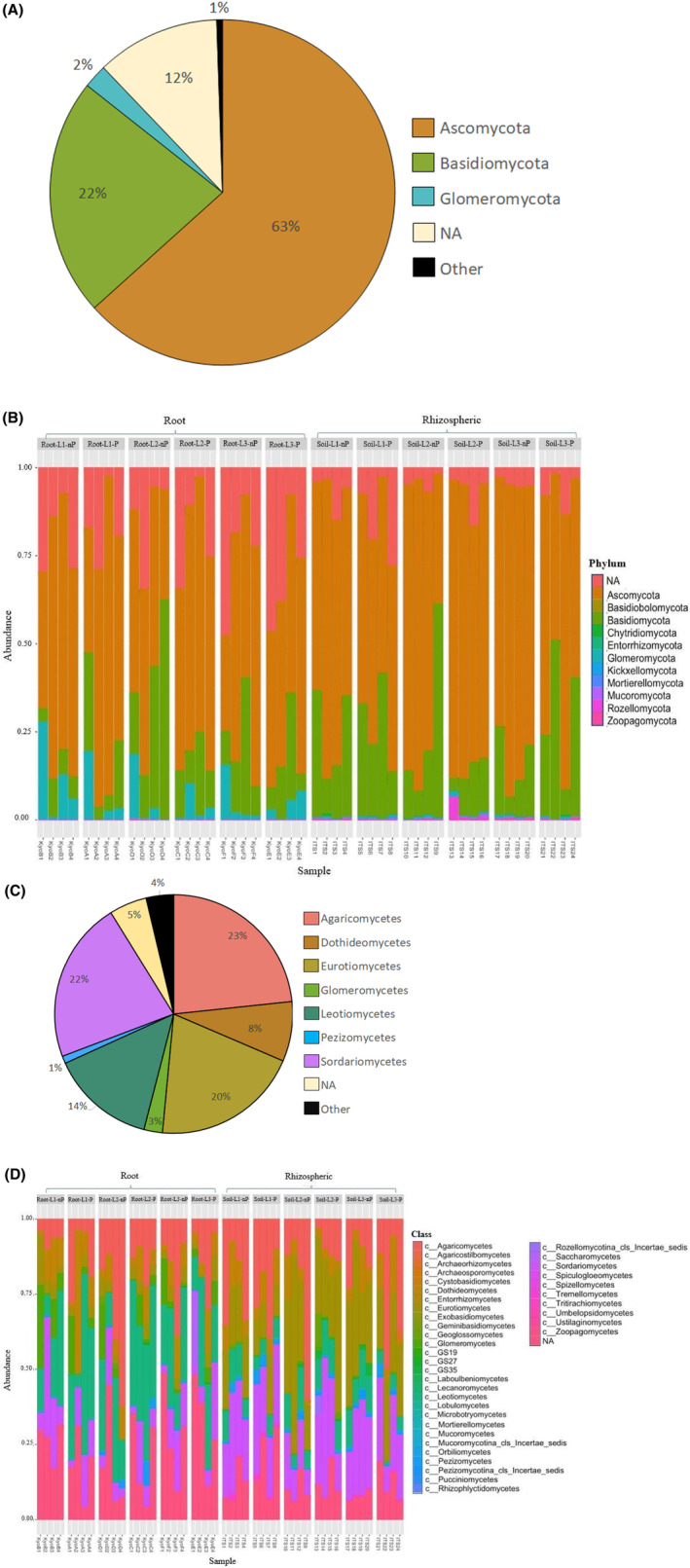
Taxonomic distribution patterns of the fungal population at phylum and order levels: Distribution of the global fungi community at phylum level (A) and across the major classes (C). Relative abundance profile (percentage of total ITS gene sequences) at the phylum level (B) and across the major classes (D) of ITS gene amplicon data, for each soil and root samples at each sampled site. Relative abundance was computed from the number of fungal ITS region amplicons. “NA” category: ASVs that did not obtain taxonomic assignment at phylum or class level; “Other” category: Mix of the classes of low abundance (<1%).

From the amplicons obtained with the primers targeting the fungal ITS region, non‐assigned ASVs to a phylum represented about 12% of the community in terms of relative abundance. Ascomycota was by far the most prevalent fungal phylum (63%) while Basidiomycota represent 22% of all the community profile (Figure 6A,B). Agaricomycetes (23.3%), Sordariomycetes (21.9%), Eurotiomycetes (20%), Leotiomycetes (14.2%) and Dothideomycetes (8.1%) are the major classes observed, whereas NA at class level reached 5.1%. The major orders are Helotiales (13.6%), Chaetothyriales (9.9%), Hypocreales (9.1%), Agaricales (8.3%), Sebacinales (8.2%), Eurothiales (8%) and Pleosporales (6.3%) with 14.6% of NA (Figure 6C,D). Only about 2% of ASVs were affiliated to the phylum Glomeromycota, and only arbuscular mycorrhizal fungi inferred to the Glomerales order, then to the Claroideoglomeraceae and Glomeraceae families, were identified using the ITS3_KYO2/ITS4_KYO3 PCR primers.

Overall, the dominance of bacterial phyla Actinobacteriota, Proteobacteria and fungal phyla Ascomycota and Basidiomycota persisted in all samples as well as across all sites (Figures 5B,D; and 6B,D).

### Taxonomic distribution between root and rhizospheric compartments

In root samples, we noted 26 bacterial phyla present, with Actinobacteria (70%), Proteobacteria (21%), Bacteroidota (4.7%) and Myxococcota (1.5%) having the highest relative abundances. Concomitantly, in soil samples, 24 different phyla were observed with Actinobacteria (70%), Proteobacteria (20%), Myxococcota (2.8%), Gemmatimonadota (2.3%) and Acidobacteriota (1.47%) being the most abundant.

Comparison of the bacterial composition across soil versus root samples indicated significant differences at different taxonomic levels (Metabarcoder differential abundance analysis with wilcox_*p*_value <0.05). Thus, the relative abundance of Actinobacteriota, Acidobacteriota, Myxococcota, Gemmatimonadota, Verrucomicrobiota and alpha‐Proteobacteria classes was significantly higher in the rhizosphere compared to the roots while the proportion of Bacteroidota and Chloroflexi were significantly increased in root compared to rhizosphere‐compartment (all localizations pooled). At order level, bacteria identified as Streptomycetales, Cytophagales, Flavobacteriales and Pseudomonadales were enriched in roots.

In the fungal data set, 11 and 8 phyla were found, respectively, in soil versus root samples. Increased relative abundances of Ascomycota was observed in soils compared to roots, with a prevalence of Sordariomycetes, Dothideomycetes and Eurotiomycetes at class level. Similarly, preferential presence of Basidiomycota in the rhizosphere was observed. At class level, reads annotated as Agaricomycetes were significantly overrepresented in rhizosphere compartments. Interestingly, within the Glomeromycota phylum, the relative abundance of members of the Glomerales order was significantly higher in roots than in the rhizosphere.

### Inputs of RP: Effects in the higher microbial taxa ranks

Comparison of the bacterial composition across P‐soils and nP‐soils were performed at different taxonomic levels. No differential response was accurately established, neither at bacterial phylum, class or order taxonomic ranks, in any localization according to the Wilcoxon Rank Sum test (wilcox_*p*_value >0.05). Likewise, no variations in abundance were measured in the fungal community, when analysing the pairwise comparison of P‐soils and nP‐soils, whatever the location and the taxonomic ranks (from phylum to order) examined. Despite the taxonomic distribution as a function of plant species was beyond the scope of this article, trends in beta diversities as well as in bacterial and fungal taxonomic distributions in roots and rhizospheric soil as a function of the P level and the plant species are provided in Figure S3.

### Microbial ASVs indicators of P‐soils

The predictive values of species as indicators of P versus nP sites as clustering groups, possibly interacting with root versus soil habitats, was calculated using a multilevel pattern analysis. Based on the indicator value calculation as a measure of specificity and fidelity to a group, a set of indicator species associated to each environmental condition were identified (Table S3). Indicator AVSs of P‐soil accounted for 3.1% and 1.4% of all AVSs, for the bacterial and fungal community, respectively. Of these, 50 and 13 ASVs were associated with the root compartment, for bacteria and fungi, respectively, while 130 and 76 originated from the soil compartment, for bacteria and fungi, respectively.

Looking at the taxonomic assignment (Table S3) and mean relative abundances of these indicator taxa, we found that bacterial indicator ASVs affiliated to at least 15 phyla, with the majority of sequences belonging to Actinobacteriota (74%) and Proteobacteria (19.4%). At class level, most ASVs were classified in Actinobacteria (46.7%), Alphaproteobacteria (14.7%), Thermoleophila (13.7%), Acidimicrobiia (8.8%) and Gammaproteobacteria (4.7%). For fungi, indicator ASVs were distributed across seven phyla, with most sequences belonging to the Ascomycota (68.7%); only 10.8% and 1.5% of indicator ASVs were affiliated to the Basidiomycota and Glomeromycota phyla, respectively. Four other phyla were also recorded, each represented by only 1 or 3 ASVs: Chytridiomycota (3), Rozellomycota (3), Mucoromycota (1) and Zoopagomycota (1). Within the phylum Ascomycota, the class Sordariomycetes is dominant and, overall, the 99 fungal indicator ASVs belonged to 20 different orders, most of them identified as Hypocreales or Helotiales.

Considering bacterial indicator species closely related to P sites (44 iASVs while 94 iASVs were obtained for nP site), we found the highest number of potential indicator taxa within the Actinobacteria (16 iASVs) and Thermoleophilia (11 iASVs) classes (Table S3). Representatives of orders Frankiales, Rhizobiales, Burkholderiales were also observed, as well as of Gaiellales and Solirubrobacterales (8 iASVs). Interestingly, iASVs assigned to the genus Streptomyces (ASV56), Bacillus (ASV564), Mycobacterium (ASV144) or Agromyces (ASV143) were listed.

Most classes to which iASVs are assigned were shared by both P and nP sites. However, for each class, iASV numbers and relative abundance (i.e., sequence number) tends to be lower in the P clustering group compared to nP group.

For fungi (Table S3), indicator species closely related to P sites (28 ASVs while 31 iASVs were obtained for nP site) included mainly representatives of classes Sordariomycetes (6 ASVs) and Leotiomycetes (8 ASVs, all inferred to Helotiales order).

The Eurotiomycetes class was not observed in P sites, but was present in nP sites. Considering abundance, Agaricomyctes are significantly dominant in P sites, as well as Dothideomycetes and Leotiomycetes to a lesser extent.

## DISCUSSION

Generally, P is present in only trace amounts in the Earth's crust (0.09 wt %) and the total P content in soils typically ranges from 50 to 3000 mg kg^−1^ depending on parent material, vegetation cover and management history (Filippelli, 2008; Frossard et al., 2004; Rai et al., 2013; Sanyal & De Datta, 1991). In this study, using an experimental setup distinct from those typically used in literature to assess P as a driver of soil microbiome, we explored microbial communities in soils with total P concentrations ranging from 1067 to 13,928 mg kg^−1^, and available Olsen P ranging from 5 to 339.5 mg kg^−1^. Our aim was to investigate how microbial community metrics such as alpha and beta diversity, taxonomic composition, and indicator taxa could inform us about the bacterial and fungal microbiomes of soils characterized by high P concentrations. These high P levels resulted from local soil enrichment with RP due to past mining activities and currently observed following natural ecosystem recovery. We compared the microbial communities of sites characterized by abandoned mining deposits with those adjacent reference sites that had no previous deposits. This comparison allowed us to assess how the high P levels, resulting from long‐term soil RP disturbances, influence the soil microbial communities in spontaneously restored sites.

### Tracking P as an edaphic driver of the current microbial communities requires to perform analysis with a stringent level of taxonomic resolution

Understanding how microbial communities are assembled in soil and which fundamental processes are shaping them is a challenging and important topic in microbial ecology. In this study, soil compartmentalization into rhizosphere or root habitats emerged as a key factor influencing microbial diversity, with distinct bacterial and fungal communities identified in each plant‐associated compartment. We observed higher bacterial alpha diversity in the rhizosphere compared to roots, regardless the metrics used and whether the analysis was global or conducted separately within each localization (L1 or L2 or L3). Regarding the global fungal diversity, Shannon indices values were also significantly higher in soil compared to root compartment. Moreover, our data highlighted that compartments exert a significant effect on beta diversity for bacteria and fungi, consistently with literature data about P‐supplemented soils (Fabiańska et al., 2019; Gómez‐Muñoz et al., 2018; Robbins et al., 2018).

In addition to soil compartmentalization, edaphic factors are also well‐known to cause significant variations in community profiles. Notably, substantial evidence in the literature documents that P disturbances in soils co‐occur with various shifts in soil fungal or bacterial communities, such as changes in diversity, taxonomic composition, relative abundance of certain microbial taxa, or functional profiles. Therefore, in our study, we also investigated how different P concentrations, resulting from the past RP enrichment on soil surfaces, influenced the current soil microbial communities. We hypothesized that differences could be observed across the different soil microbiomes we sampled. However, because no clear consensus exists in the literature regarding key taxonomic groups responsive to P inputs, our analysis was performed at various community and taxonomic levels.

Firstly, our data highlighted that alpha diversity metrics do not consistently show significant differences when comparing P‐enriched soils to non‐P‐enriched soils across various locations, despite the markedly different P contents. However, some variation in microbial beta diversity indices was observed according to P status, depending on location and bacterial or fungal communities. For their part, Robbins et al. (2018) concluded that P had little effect on alpha and beta diversity after extended exposure to long‐term contrasting fertilization regimes. Conversely, Silva et al. (2017) found that long‐term fertilization with RP and triple superphosphate resulted in significantly higher bacterial Shannon indices in RP‐treated samples compared to the control.

Interestingly, our data also showed that the different microbial communities displayed similar patterns in terms of dominant taxa identity, regardless of past RP input events or current soil P concentration. The bacterial phyla Actinobacteriota, Proteobacteria and the fungal phyla Ascomycota and Basidiomycota, were dominant in all sampling sites, irrespective of P concentration. These results suggest that drastic variations in P levels do not induce significant changes in the overall current microbial community composition. Minor shifts in soil bacterial and fungal communities were also recorded by Wakelin et al. (2012) and Wang et al. (2016) in studies on long‐term effects of P.

In contrast, Trabelsi et al. (2017) observed short‐lived effects of P fertilization that were significant: members of Gammaproteobacteria and Bacteroidetes were stimulated by high RP level s (250 kg P ha^−1^) while Firmicutes decreased. Conversly, members of Betaproteobacteria and Actinobacteria increased with RP fertilization at low P levels (i.e., 50 kg P ha^−1^). Previously, Wakelin et al. (2012) hypothesized that soil Actinobacteria could be linked to P status. Comparing different P‐fertilization treatments, Silva et al. (2017) noted that Proteobacteria was the predominant phylum in the microbial community of the maize rhizosphere regardless of the P treatments. Additionally, they observed that while Enterobacteriaceae taxa (Gammaproteobacteriacae) decreased with RP, Oxalobacteraceae (mainly *Massilia* and *Herbaspirillum*) and Burkholderiaceae increased with RP addition. Bacillaceae also showed significantly higher abundance in RP soils. Finally, *Burkholderia* sp. and *Bacillus* sp. were enriched, and *Klebsiella* was the second most abundant taxon in the RP‐treated soil. In conclusion, our results confirm that the microbial response to changes in P content is highly variable in terms of shifts in taxonomic composition.

Finally, despite the conclusion that microbial community profiles at coarse taxonomic scales were robust across the contrasted P levels of sampled soils, we explored whether key ecological insights could be gained from finer‐scale analyses. To this end, we performed a multipatt indicator value analysis to identify indicator species specifically associated with P‐rich soils. This approach clearly differentiated P and non‐P sites, highlighting that bacterial and fungal communities are responsive to P as an edaphic factor when analysed with a high level of taxonomic resolution.

### Explaining the microbial response upon exposure to long‐term high P pressure by a legacy effect of historical contingencies and microbial life strategies

In our study, the RP input is characterized as a one‐time pulse of nutrient P resulting from a severe and detrimental ecological disturbance caused by RP ore deposits on the soil surface due to mining activities. This was followed by over a century of natural and gradual whole‐ecosystem restoration, during which high phosphorus levels have persisted. Across this study, large similarities have been recorded across the sampled microbial assemblages in terms of prevailing taxa, namely Actinobacteriota and Ascomycota, as well as Proteobacteria and Basidiomycota to a lesser extent. Despite P being recognized in the literature as a potential driver of microbial community composition, we were surprised to find minimal changes in soil microbial community patterns even when there were significant differences in P levels.

Here, literature data highlight that past events may have long‐termed legacy effects on community composition; these are referred to as historical contingencies (Fukami, 2015). However, to which extent historical contingencies shape microbial assembly processes in natural ecosystems is still poorly documented and it remains an open question in microbial ecology. Nevertheless, they may prove critical in attempting to unravel the P effect we have observed in contemporary communities. In combination, although it is generally difficult to infer the ecological function of a microbe solely based upon a taxonomic assignment, it may be relevant to examine ecological significance of the dominance of specific taxa, and how microbial life strategy concepts (i.e., copio‐ and oligotrophic concepts; Fierer, 2017; Ho et al., 2017) and the ecological traits of the dominant microbiological taxa could explain, more than soil P contents, the prevalence of few taxa and homogeneity in microbial patterns across soil samples studied. Indeed, microorganisms are physiologically diverse, possessing disparate genomic features and mechanisms for adaptation (functional traits), which reflect on their associated life strategies and determine at least to some extent their prevalence and distribution in the environment. In our study, among the prevalent phyla, we note many taxa referred to as copiotrophic taxa according to the oligotrophic/copiotrophic theory. Notably, Betaproteobacteria, known to be more abundant in organic carbon‐rich habitats, have been generally classified as copiotrophic microbes (Fierer, 2017; Francioli et al., 2016; Ho et al., 2017). In contrast, the trophic categorization of Actinobacteria remained unclear thus far, and mixed results about their response to nutrient status have been documented, with some results reporting a copiotrophic status with an increase in abundance with nutrient contents while others reported no change. Nevertheless, they are generally viewed as being ubiquitous and widely distributed across terrestrial ecosystems (Delgado‐Baquerizo et al., 2018; Lewin et al., 2016; Sayed et al., 2020; van Bergeijk et al., 2020). They are also considered as important contributors to the process of plant biomass decomposition due to their cellulolytic enzymes. Moreover, although Actinobacteria are still generally viewed as free‐living bacteria with a saprotrophic lifestyle, their common association with plants, including in the rhizosphere and as endophytes is well established (Chen et al., 2019). In addition, Actinobacteria and Proteobacteria are known as copiotrophic microbes which display faster growth rates under high C availability conditions. In opposite, Acidobacteria are typically referred to as oligotrophic microorganisms, which tend to exist in nutrient‐deficient and strongly acidic environments (Cui et al., 2021; Yao et al., 2017).

The oligotrophic/copiotrophic theory is less frequently used to discuss the ecological roles of dominant fungal taxa. However, it is suggested that saprotrophic fungi may exhibit copiotrophic features (Yao et al., 2017) and Ascomycota phylum which include important decomposers of organic substrates and many wood‐decay saprotrophs, notably the Eurotiomycetes, Leotiomycetes and Dothideomycetes (Lundell et al., 2014) are classified into the copiotrophic categories. Ascomycota has been found to be a prevalent fungal phylum in various environments due to their diversity in terms of metabolic capacities, owing to the wide variety of enzymes they produce. In contrast, many members of the fungal phylum Basidiomycota (for which a lower quantity of sequences was recorded in our samples) may predominantly behave as oligotrophs despite this phylum also hosts fungal decomposers of plant litter with high lignin content (Ma et al., 2013) indicating copiotrophic traits.

All together, these data are in accordance with our sampling conditions. Firstly, we exclusively sampled root and rhizosphere compartments. Across such soil compartments, the dynamics of plant microbiome acquisition and profiling remain an ill‐understood complex process (Fitzpatrick et al., 2018; Peiffer et al., 2013). Nevertheless, according to the classical models, the rhizosphere is described as a nutrient‐rich compartment (Hartman et al., 2018) attracting only a subset of microbes present in the bulk soil thanks to qualitative and quantitative patterns of root exudates (rhizodeposition) and thus, orchestrating the composition and function of microbial populations (Marschner et al., 2004; Philippot et al., 2013; Tkacz et al., 2020). In turn, the rhizoplane and the endoderm are supposed to impose a selective filter that controls further microbial colonization inside the root compartment and depletion of rhizospheric taxa into the endosphere (Bulgarelli et al., 2013; del Carmen Orozco‐Mosqueda et al., 2018; Hartman et al., 2018). Concomitantly, the root endodermis delimits a protective biotope, also with rich‐nutritional characteristics. Consequently, being classically described as carbon‐rich hotspots, root and rhizosphere compartments can be referred to as copiotrophic biotopes.

In addition, we can hypothesize an accumulation of organic matter and organic carbon during ecosystem restoration of the sample sites, due to the absence of anthropogenic disturbance or export of plant material for more than one century. Therefore, these ecosystems could also be compatible with the growth of copiotrophic populations in the bulk soil and upper soil layers, that is, with a high abundance of Actinobacteria and Ascomycota among the most notable taxa observed in plant‐associated compartments. However, although many studies have suggested that Actinobacteria and Betaproteobacteria are (sub)phyla associated with copiotrophy, other studies that consider finer levels of taxonomic or phylogenetic resolution (e.g., family, genus and species level), suggest that some members of these phyla are better adapted to oligotrophic conditions. Consequently, the question of whether microbial life strategy concepts alone can explain, the prevalence and distribution of some microbial communities remain open as discussed by Ho et al. (2017).

The homogeneity in microbial patterns observed in our study could be explained through the respective contributions and prevalence of the deterministic versus stochastic processes, two major ecological processes, proposed to explain the spatial patterns of soil microbial diversity (Caruso et al., 2011; Deakin et al., 2018; Vellend et al., 2014; Zhang et al., 2016). We hypothesize that deterministic processes operated immediately after the drastic mining disturbance, inducing the environmental filtering of microbial generating microbial dissimilarity between P and nP soils. Then, after decades of soil restoration subsequently to mining inputs, both bacterial and fungal communities of disturbed soils have reached patterns quite comparable to those of non‐disturbed soils, due to the prevalence of stochastic dispersal processes known to determine the similarity of microbial communities, notably at small spatial scales, in neighbouring sites (Deakin et al., 2018).

Moreover, plants shape the recruitment and assemblages of soil microbiota (Bulgarelli et al., 2013; Fitzpatrick et al., 2018; Klasek et al., 2021; Leff et al., 2018; Thiergart et al., 2020; Trivedi et al., 2021). In our study, the sampled plants, representatives of extensively different phylogenetic species, are known to be widely distributed in numerous habitats, adapted to a large range of physical and chemical soil properties, also including ecosystems strongly disturbed by anthropogenic activities. The sampled plants also resulted from evolutionary adaptation during the natural ecosystem restoration process of the disturbed mining sites, coping with abiotic P‐stress.

As plant species have a pivotal effect on the rhizosphere and root microbiome, it can be hypothesized that the observed dominant microbial taxa may reflect the influence of ecological plant traits, including the co‐evolution of host plants with root microbiota, mitigating abiotic environmental stressors (such as the P level) experienced by host plants (Fitzpatrick et al., 2018). In this study, it appears that the host plant may have a more significant influence on their associated microbiomes than soil characteristics over a long period.

### Microbial communities of mining sites as a resource for agriculture and land restoration

Both abundant and rare microbial species in soil are important members of the microbiota and should be considered important for agriculture and land restauration.

We hypothesize that RP mining soils harbour microbial communities adapted to local P‐enriched conditions. Consequently, these soils may provide a unique ecological reservoir of microbial resources, where key taxonomic groups responsive to P inputs and essential taxa for ecosystem functioning could be identified for further agronomic applications. This hypothesis is supported by previous findings that report bacteria from mining sites with multiple plant growth‐promoting traits, particularly P‐solubilization abilities (Ducousso‐Détrez et al., 2024). Such microorganisms could be relevant for the production of biological inoculants in agronomic research, especially for soils enriched in P due to past agronomic practices and chemical inputs. Additionally, these strains could play a decisive role in land reclamation, serving as key resources and major assets for reforestation and revegetation programs aimed at rehabilitating mine sites.

Given that Actinobacteriota and Ascomycota were the dominant soil microbial phyla in our study, we propose that future research should focus on taxa from these prevalent communities. Specifically, the dominant Actinobacteria taxa present an intriguing avenue for investigation. Actinobacteria are ubiquitous in various ecosystems and are characterized by adaptive functional traits such as sporulation, competitive life strategy, and low sensitivity to environmental stress (van Bergeijk et al., 2020). Several taxa within this group have been found to enhance plant fitness, with examples such as Streptomyces known to improve stress tolerance.

We have also identified indicator ASVs of P‐enriched sites, providing insights into the microbial composition adapted to high‐P abiotic conditions. These taxa warrant further investigation to characterize their potential beneficial effects on plants and their utility as restoration tools in the future. Notably, our analyses highlighted different taxonomic groups known in the literature to host phosphate‐solubilizing bacteria, such as *Streptomyces*, *Bacillus*, *Mycobacterium* or *Agromyces* or bacteria assigned to Rhizobiales and Burkholderiales (Alori et al., 2017). Cultivation of these taxa could be of interest to evaluate their phosphate solubilizing ability. Utilizing these taxa as bio‐inoculants in conjunction with RP fertilizer could promote more sustainable agricultural practices, particularly since excessive P fertilization is unfortunately common.

## CONCLUSION AND PERSPECTIVES

The ecological importance of microbial communities in the ecosystem functioning is now well recognized throughout the literature. Our study stands out as one of the few conducted in a naturally restored mining environment, laying the groundwork for a more detailed understanding of the relationship between phosphorus, microorganisms, and plants. Future research endeavours should focus on isolating taxa of interest and obtaining functional information about microbial communities in RP‐rich sites.

## AUTHOR CONTRIBUTIONS

**Amandine Ducousso‐Détrez:** Conceptualization (equal); data curation (lead); formal analysis (lead); investigation (lead); methodology (lead); validation (equal); writing – original draft (lead). **Simon Morvan:** Data curation (supporting); formal analysis (supporting); methodology (supporting); validation (equal). **Joël Fontaine:** Conceptualization (equal); funding acquisition (equal); project administration (equal); resources (equal); supervision (equal); validation (equal). **Mohamed Hijri:** Conceptualization (equal); funding acquisition (equal); investigation (equal); project administration (equal); resources (equal); supervision (equal); validation (equal). **Anissa Lounès‐Hadj Sahraoui:** Conceptualization (equal); funding acquisition (equal); investigation (equal); project administration (equal); resources (equal); supervision (equal); validation (equal).

## CONFLICT OF INTEREST STATEMENT

The authors declare no conflict of interests.

## Supporting information


**DATA S1.** Supporting Information.

**TABLE S3.** Bacteria and fungal indicator species–excel format. Bacterial and fungal indicator species (ASVs) performing a multilevel pattern analysis considering the P versus nP sites as clustering groups (A, B) or a multilevel pattern analysis considering P versus nP sites, and root versus soil habitats as clustering groups and considering all possible combinations of the sites/habitats (C, D).

## Data Availability

The dataset supporting the conclusions of this article is available in the NCBI Sequence Read Archive (SRA) database and can be found under the project accession number PRJNA784523. For supplementary files: Please find the DOI link below: 10.6084/m9.figshare.26796160
