# Supplementary material for: How do high phosphate concentrations affect soil microbial communities after a century of ecosystem self‐reclamation?
Source: Environ Microbiol Rep. 2024 Oct 23;16(5):e70003. doi: 10.1111/1758-2229.70003 (PMC11497093; doi:10.1111/1758-2229.70003)
Supplement: Supplementary file 1 — DATA S1. Supporting Information. [file EMI4-16-e70003-s002.pdf]

Supplementary data

Table S1: Data from the bioinformatic processing after Illumina MiSeq sequencing

	BACTERIA	FUNGI
Nb Samples	48	48
Nb raw MiSeq reads	4,890,709<br>soil: 2,457,648<br>roots: 2,433,061	3,642,907<br>soil: 2,674,883<br>roots: 968,024
Nb reads filtered after DADA2 step	1,126,828	1,611,669
Nb reads per sample	from 9,660 to 45,661	from 825 to 127,566
Nb ASV inferred	6,720	6,664
Nb singletons<br>Nb doubletons	51<br>433	31<br>402
Nb ASV retained	5,671	6,231

Supplementary data

Table S2: Shannon and Simpson Alpha diversity of bacterial and fungal communities within the rhizospheric or root samples

48 samples harvest across 6 sites (L1-P, L1-nP, L2-P, L2-nP; L3-P, L3-nP), 2 plant-associated compartments (rhizospheric soil /root) and 4 plant species (one plant species sample being a composite sample mixed from 3 individuals)

Bacteria

		Chao1	Shannon	Simpson			Chao1	Shannon	Simpson
Root L1-P	**BA1**	439.5	5.465	0.993	Soil L1-P	**BS1**	623,375	5.959	0.996
	**BA2**	422,125	5.484	0.993		**BS2**	381	5.562	0.995
	**BA3**	189.3	4.679	0.984		**BS3**	267	5.115	0.991
	**BA4**	381.0	5.316	0.990		**BS4**	450	5.605	0.995
Root L1-nP	**BB1**	311.2	4,496	0.951	Soil L1-nP	**BS5**	602	5.967	0.996
	**BB2**	316.0	5.223	0.991		**BS6**	688	6.045	0.996
	**BB3**	421.0	5.645	0.994		**BS7**	451	5.684	0.995
	**BB4**	322.0	3.807	0.851		**BS8**	606	5,956	0.996
Root L2-P	**BC1**	319,125.0	5.143	0.989	Soil L2-P	**BS9**	436	5.609	0.994
	**BC2**	412.2	5.231	0.989		**BS10**	634	5.953	0.996
	**BC3**	239.8	4.839	0.983		**BS11**	546	5.781	0.995
	**BC4**	188.0	4.615	0.982		**BS12**	700	6.059	0.996
Root L2-nP	**BD1**	543.1	5.736	0.995	Soil L2-nP	**BS13**	640	5.944	0.996
	**BD2**	361.0	5.431	0.993		**BS14**	380	5.443	0.994
	**BD3**	449.0	5.409	0.990		**BS15**	288	5.205	0.992
	**BD4**	311.0	5.105	0.990		**BS16**	494	5.736	0.995
Root L3P	**BE1**	659.1	5.975	0.996	Soil L3P	**BS21**	500	5.865	0.996
	**BE2**	231.0	4.926	0.989		**BS22**	349	5.507	0.995
	**BE3**	383.3	5.162	0.987		**BS23**	280	5.278	0.993
	**BE4**	162.0	3.683	0.902		**BS24**	592	5.877	0.994
Root L3-nP	**BF1**	389.0	5.303	0.991	Soil L3-nP	**BS17**	668	6.009	0.996
	**BF2**	239.0	4.892	0.987		**BS18**	455	5.642	0.995
	**BF3**	386.1	5.099	0.986		**BS19**	470	5.745	0.995
	**BF4**	292.3	4.929	0.983		**BS20**	462	5.674	0.995

Fungi

		Chao1	Shannon	Simpson			Chao1	Shannon	Simpson
Root L1-P	**KyoA1**	195	3.757	0.949	Soil L1-P	**ITS1**	406.0	4.598	0.974
	**KyoA2**	217	3.667	0.922		**ITS2**	424.3	4.748	0.981
	**KyoA3**	80	3.235	0.925		**ITS3**	330.1	4.446	0.971
	**KyoA4**	397	4.369	0.963		**ITS4**	432.1	4.557	0.970
Root L1-nP	**KyoB1**	118	3.411	0.920	Soil L1-nP	**ITS5**	459.7	4.428	0.972
	**KyoB2**	188	3.919	0.958		**ITS6**	453.0	4.514	0.972
	**KyoB3**	155	4.285	0.977		**ITS7**	354.1	4.168	0.945
	**KyoB4**	197	3.699	0.920		**ITS8**	567.2	4.670	0.957
Root L2-P	**KyoC1**	137	3.607	0.946	Soil L2-P	**ITS9**	417.7	2,779	0.751
	**KyoC2**	153	3.921	0.966		**ITS10**	397.6	4.632	0.973
	**KyoC3**	131	3.765	0.954		**ITS11**	241.0	2.900	0.751
	**KyoC4**	365	4.448	0.966		**ITS12**	487.0	4.765	0.978
Root L2-nP	**KyoD1**	325	4.385	0.971	Soil L2-nP	**ITS13**	331.0	4.240	0.965
	**KyoD2**	251	4.081	0.951		**ITS14**	379.0	3.653	0.869
	**KyoD3**	144	3.613	0.946		**ITS15**	305.0	4.689	0.983
	**KyoD4**	41	2.965	0.909		**ITS16**	359.0	4.210	0.957
Root L3P	**KyoE1**	127	4.385	0.971	Soil L3P	**ITS21**	471.2	4.861	0.982
	**KyoE2**	420	4.081	0.951		**ITS22**	193.0	2.937	0.836
	**KyoE3**	193	3.613	0.946		**ITS23**	336.0	4.428	0.968
	**KyoE4**	225	2.965	0.909		**ITS24**	368.2	4.097	0.935
Root L3-nP	**KyoF1**	216	4.130	0.955	Soil L3-nP	**ITS17**	388.0	4.223	0.950
	**KyoF2**	199	4.264	0.975		**ITS18**	385.1	4.319	0.965
	**KyoF3**	68	3.608	0.954		**ITS19**	410.2	4.720	0.980
	**KyoF4**	124	3.921	0.965		**ITS20**	512.2	5.012	0.986

Supplementary data

Table S3: Bacteria and fungal indicator species– excel format

Bacterial and fungal indicator species (ASVs) performing a multilevel pattern analysis considering the P *versus* nP sites as clustering groups (A, B) or a multilevel pattern analysis considering P *versus* nP sites, and root *versus* soil habitats as clustering groups and considering all possible combinations of the sites/habitats (C, D).

Supplementary data

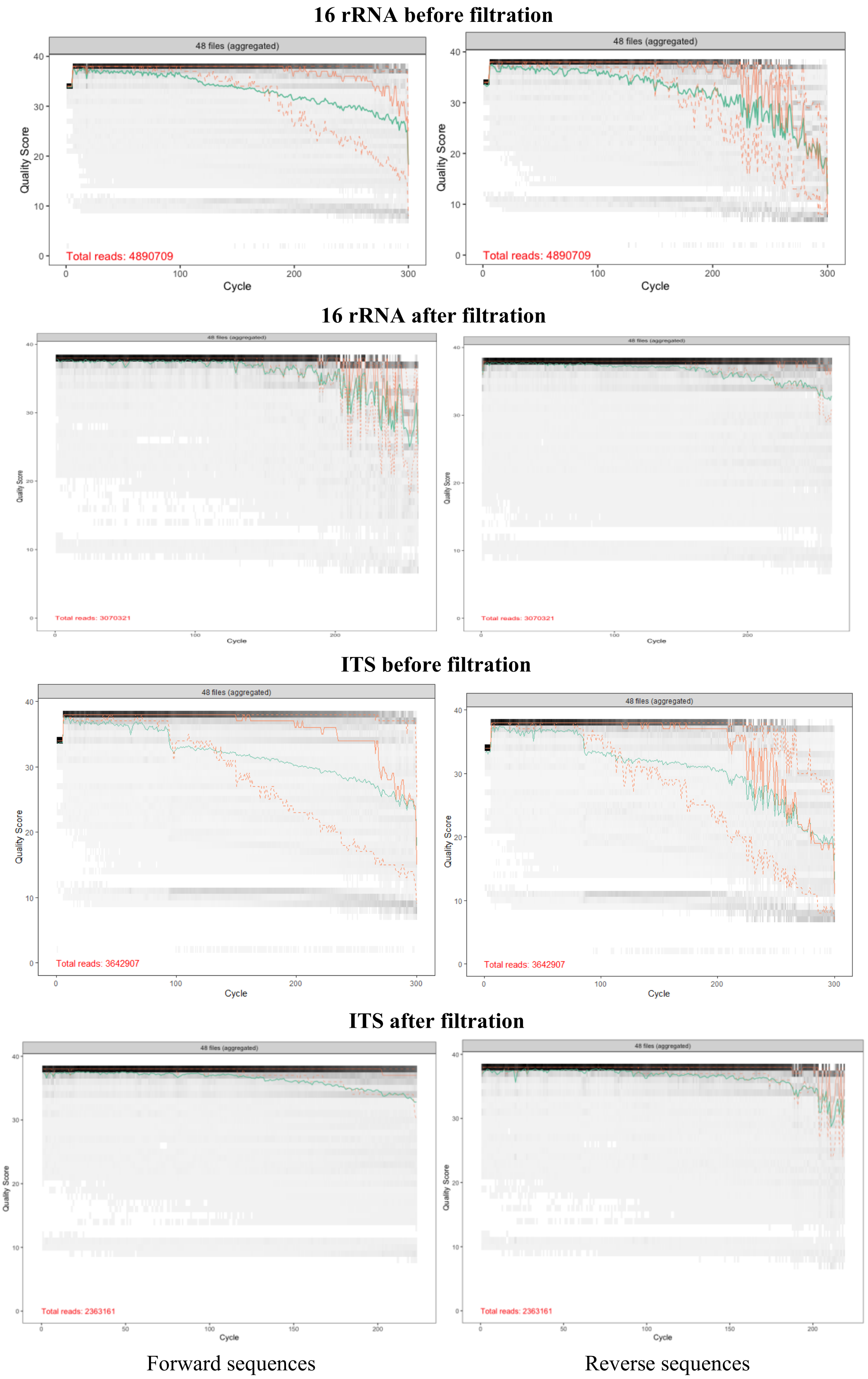

Fig. S1: Quality profiles for 16 rRNA and ITS sequences

A

Supplementary data

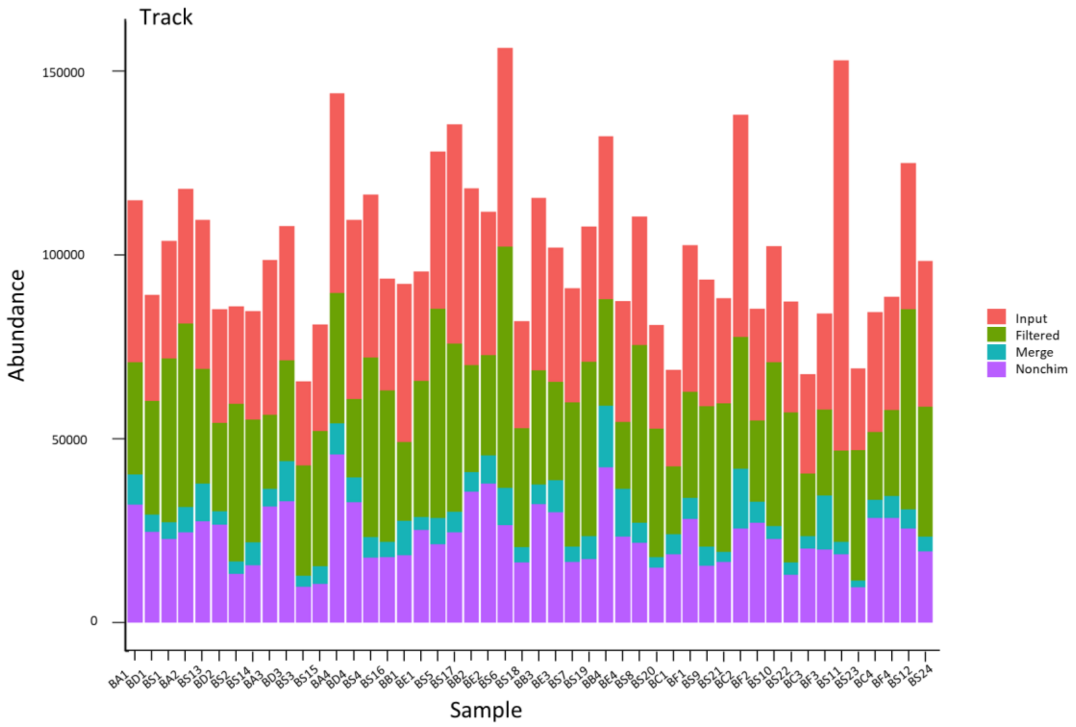

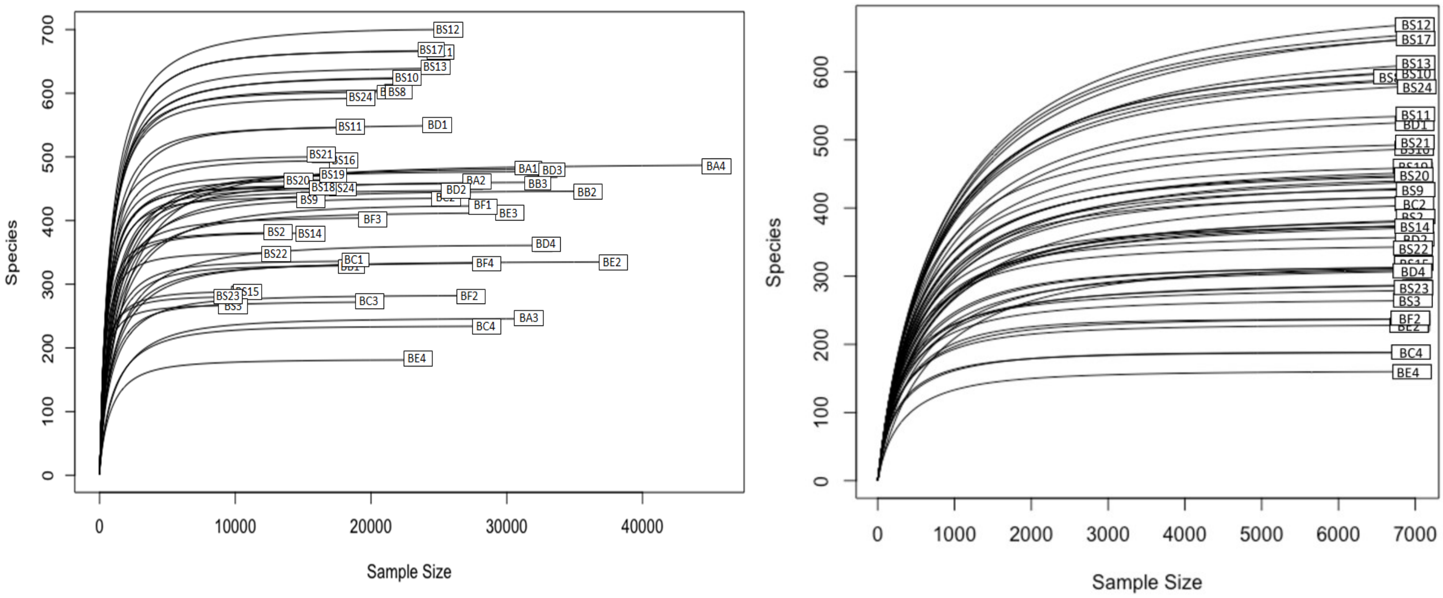

B

Supplementary data

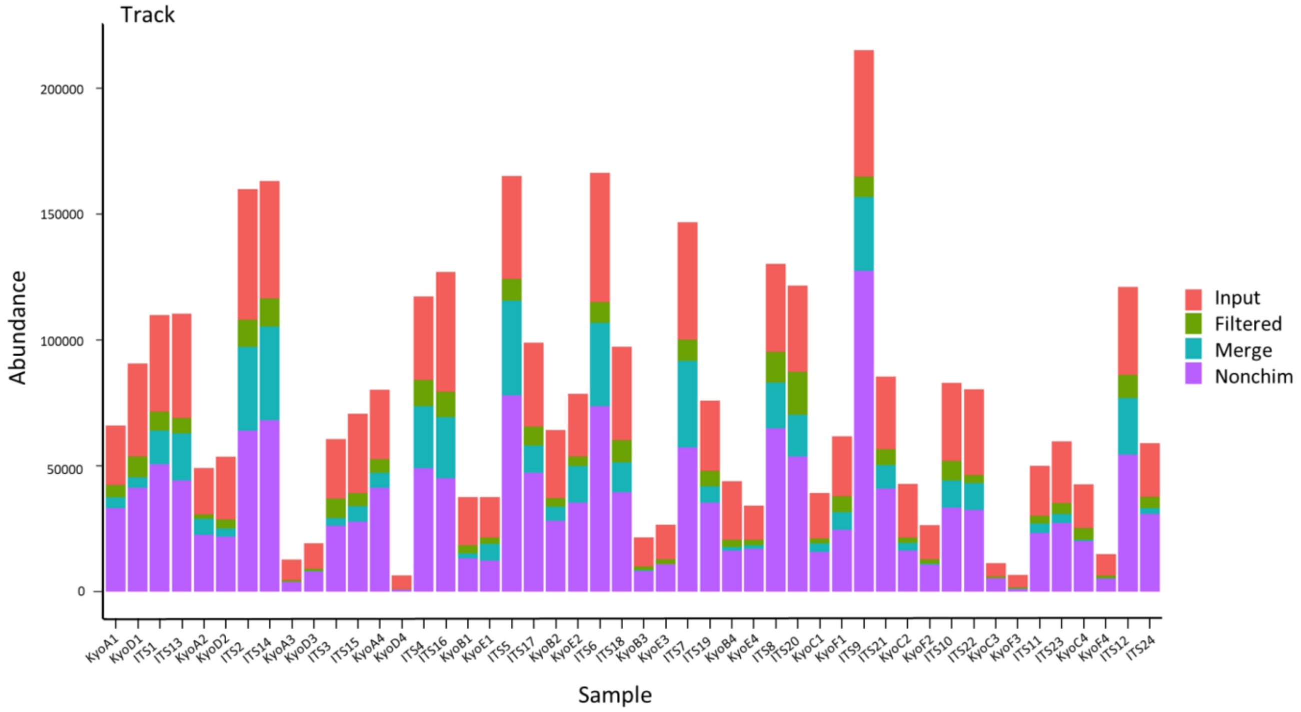

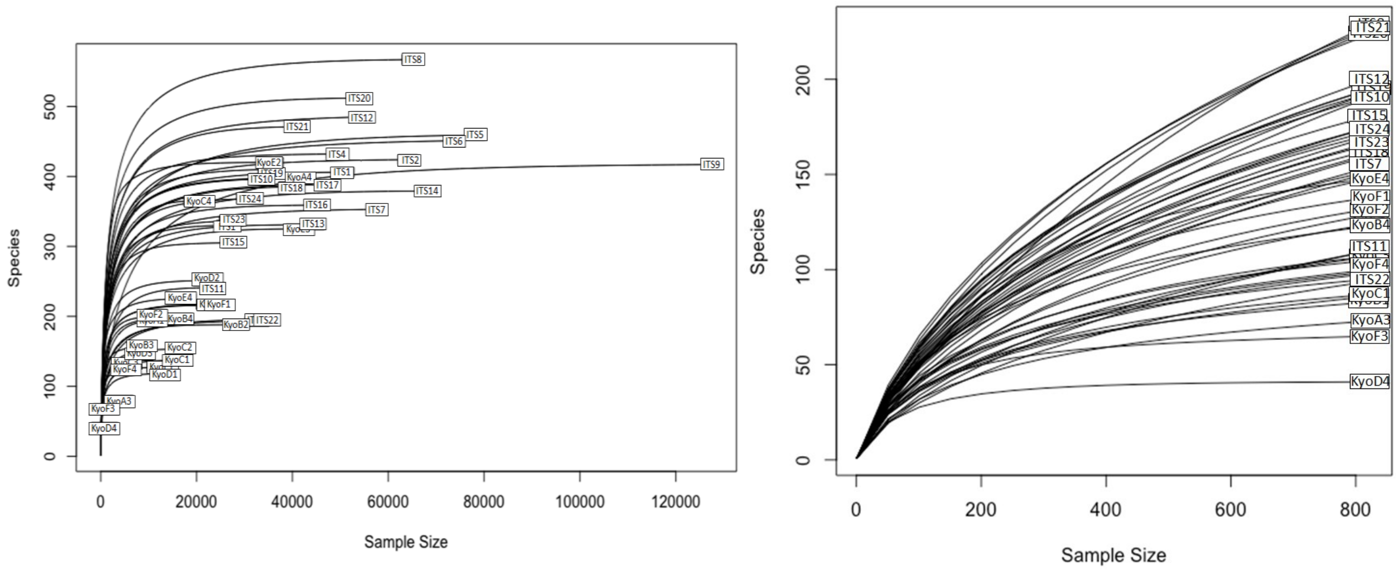

Fig. S2: Rarefaction curves for 16S rRNA (A) and ITS (B) data sets

Supplementary data

A : Bacterial beta diversity

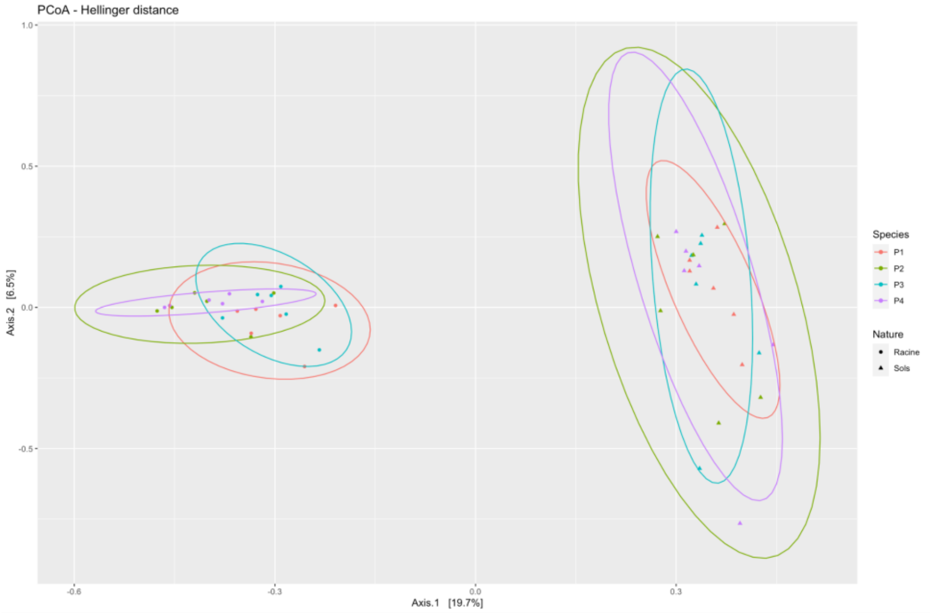

B : Fungal beta diversity

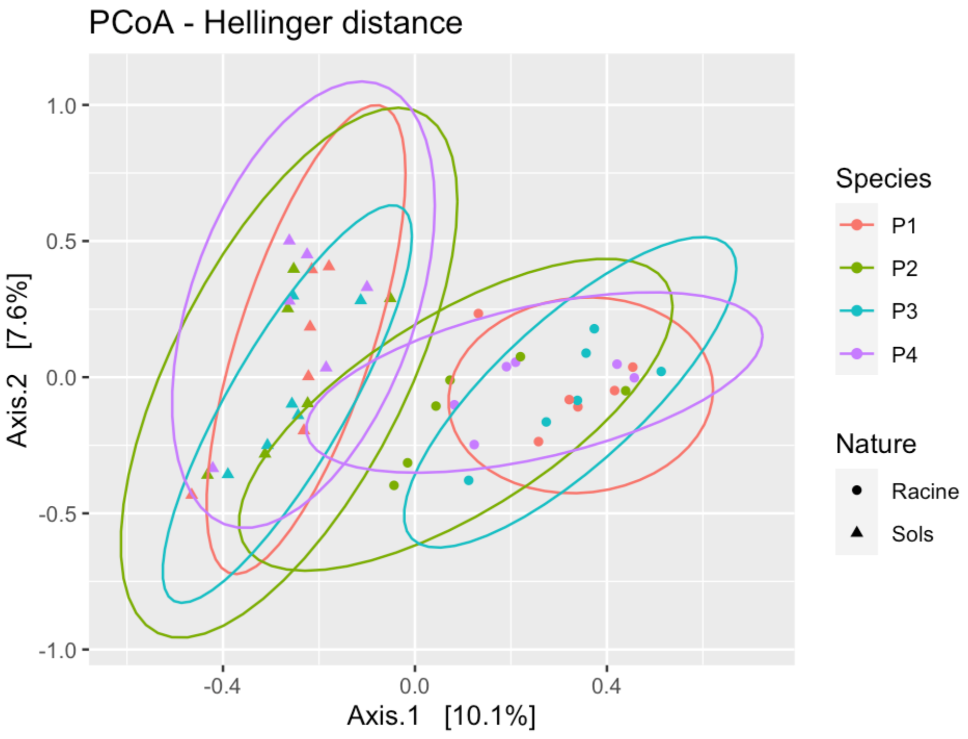

PCoA ordination, based on Hellinger distances, of bacterial 16S rRNA gene sequence data (A) and fungal ITS sequence data (B)

C : Bacterial taxonomic distributions in roots and rhizospheric soil as a function of the plant species and of P/nP sites

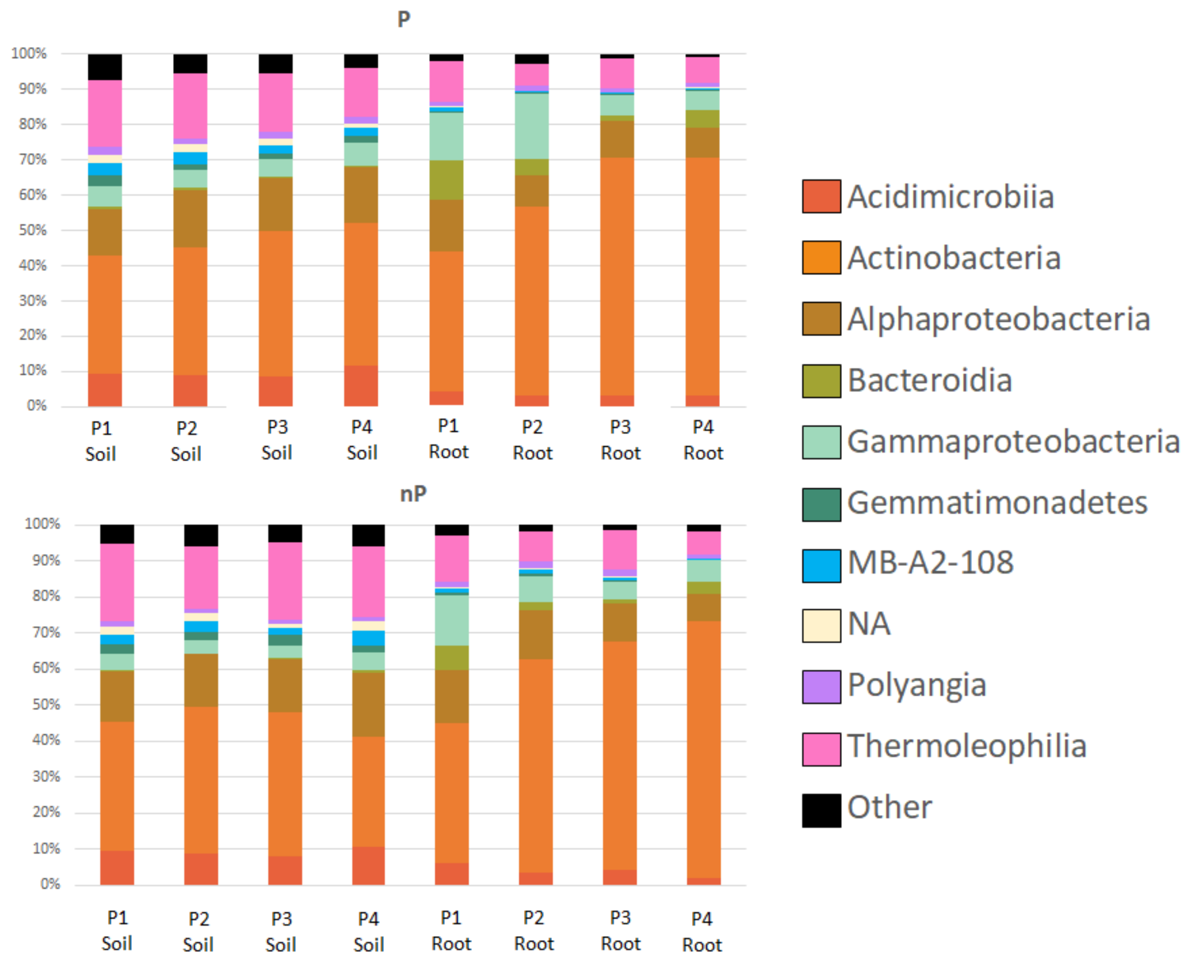

Supplementary data

D : Fungal taxonomic distributions in roots and rhizospheric soil as a function of the plant species and of P/nP sites

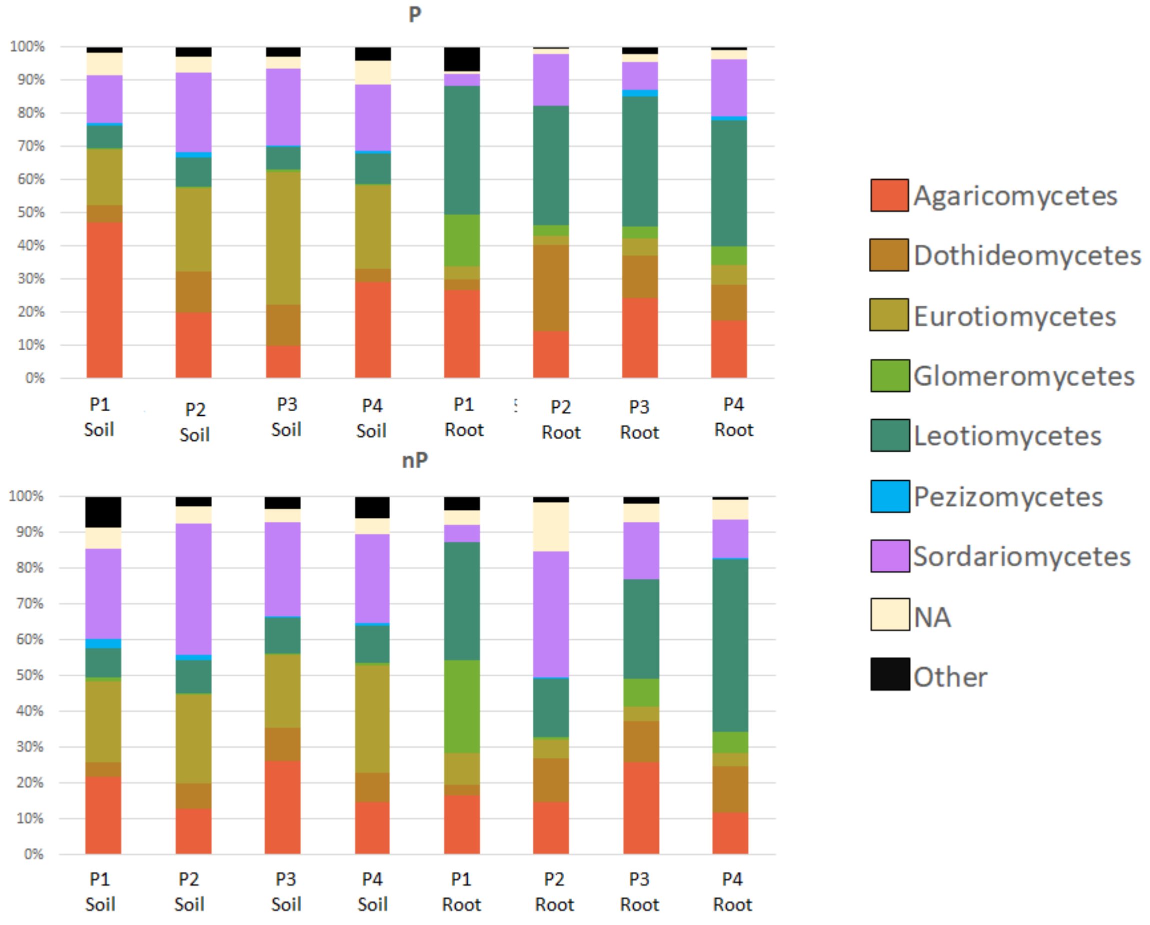

Fig. S3 : Bacterial and fungal beta diversity and taxonomic distributions, at class level, in roots and rhizospheric soil as a function of the P/nP sites and of the plant species

P1 : *Ranunculus bulbosus* L.
P2 : *Taraxacum officinale* F.H. Wigg.
P3 : *Dactylis glomerata* L.
P4 : *Bromus sterilis* L.
P: with mining RP ore deposit; nP: without mining RP ore deposit.
